# Respiratory Monitoring in Motion: An Overview of Wearable Methods and Algorithmic Approaches for Reliable Assessment

**DOI:** 10.3390/bios16060306

**Published:** 2026-05-23

**Authors:** Michal Pecik, Erik Vavrinsky, Diana Vitazkova, Helena Kosnacova, Juraj Nevrela, Erik Foltan

**Affiliations:** Institute of Electronics and Photonics, Faculty of Electrical Engineering and Information Technology, Slovak University of Technology, Ilkovicova 3, 81219 Bratislava, Slovakia; diana.vitazkova@stuba.sk (D.V.); helena.kosnacova@stuba.sk (H.K.); juraj.nevrela@stuba.sk (J.N.); erik.foltan@stuba.sk (E.F.)

**Keywords:** respiration, wearable, motion robustness, chest wall motion, derived respiration, sensor fusion

## Abstract

Advances in wearable device and sensor technologies progressively shift respiratory monitoring from the clinical setting to real-world conditions. This rapidly developing field allows for more accurate diagnostics. However, reliable monitoring during dynamic activities remains challenging due to artifacts caused by movement, postural changes, electrode drift, and variability in breathing patterns. Therefore, this review focuses on wearable methodologies capable of determining respiratory rate and potentially tidal volume during strenuous physical activities. Direct sensing approaches, including chest and abdominal belts, bioimpedance principles, and inertial sensing units, are complemented by indirect methods derived from ECG and PPG signals. Hybrid systems, which are also discussed, represent a very promising approach. Special attention is paid to signal processing, machine learning, and multimodal sensor fusion algorithms that improve robustness and reliability. By systematically analyzing hardware and software combinations, validation protocols, and current limitations, this article identifies emerging trends in adaptive respiratory monitoring. This review aims to guide the development of next-generation wearable systems.

## 1. Introduction

Respiratory rate (RR) and tidal volume (V_T_) are fundamental indicators of physiological status and cardiopulmonary function. Unlike many cardiovascular parameters, respiratory signals respond rapidly to changes in workload and exertion, providing early insights into physiological stress and adaptation. Changes in respiratory dynamics may even precede changes in heart rate or oxygen saturation. Previous studies have shown that respiratory activity is predominantly regulated by rapid neural inputs, including central command signals and afferent feedback from working muscles, while metabolic stimuli contribute to a delayed response [1,2,3]. This multitemporal regulation explains the strong association between respiratory signals and exercise intensity across a wide range of conditions. Emerging evidence suggests that respiratory parameters may even reflect physical exertion more consistently than traditional physiological markers, such as blood lactate, particularly in situations involving postexercise muscle damage, glycogen depletion, or metabolic disturbances [4,5,6]. In this context, the RR has been recognized as one of the most sensitive physiological markers, responding not only to physical effort and exercise-induced fatigue but also to emotional stress, cognitive load, thermal stress, and various pathological conditions. Nicolo et al. [7] further emphasized that respiratory monitoring has broad implications, extending from healthcare and clinical deterioration assessment to occupational safety and sports performance optimization, while also highlighting that respiratory parameters remain substantially underutilized despite the growing availability of wearable sensing technologies. Conventional reference techniques, such as spirometry and pneumotachography, allow the direct measurement of inspiratory and expiratory flow and therefore remain the highly accurate gold standard for determining both RR and V_T_. However, the need for face masks or nasal interfaces significantly limits subjects’ comfort and mobility, alters natural breathing patterns, and limits their applicability to short-term and laboratory experiments [8].

The current status quo in respiratory monitoring is dominated by a diverse range of technologies optimized for static conditions, such as sleep analysis or home-based clinical diagnostics [9,10,11]. This ecosystem includes traditional chest straps, modern bioamplifiers utilizing thoracic impedance, and sensors capturing micro-movements through seismocardiography and ballistocardiography. Remote, non-contact solutions—ranging from radar and integrated optical fibers to thermal and RGB camera systems—have also gained prominence for their ability to monitor breathing without physical attachments. Additionally, acoustic sensing and gas analysis via specialized masks offer deep physiological insights into lung function and breathing patterns. Massaroni et al. [12] provided a physically oriented framework for contact sensing that details the transduction mechanisms underlying strain, impedance, airflow, and acoustic systems. In their subsequent review [13], the authors categorized chest wall monitoring technologies into electrical, optical, and inertial modalities, complementing the hardware analysis with state-of-the-art signal processing strategies. Together, these works confirm that robust respiratory monitoring requires a joint design of sensor physics and algorithmic extraction rather than an isolated optimization of these areas. While these modalities provide high accuracy and comfort in stable environments, they often face significant limitations under dynamic movement due to susceptibility to motion artifacts and reliance on fixed environmental infrastructure [14]. Regarding signal reliability, Monaco et al. [15] demonstrated that while modalities such as inertial measurement units (IMUs), respiratory induction plethysmography (RIP), and bioimpedance provide acceptable accuracy under static conditions, performance during dynamic movement degrades significantly. A central limitation also remains the fact that subject-specific calibration is required for quantitative estimation of V_T_, as opposed to spirometry. This issue reflects a broader challenge: movement artifacts are not just noise but biomechanically coupled signals that overlap spectrally and morphologically. This article can be considered a continuation of our previous publication “Advances in Respiratory Monitoring: A Comprehensive Review of Wearable and Remote Technologies” [14], which focused primarily on wearable and remote monitoring in relatively controlled clinical contexts, especially sleep applications. That work emphasized acoustic sensing, breath analysis, and blood gas estimation, but did not prioritize motion-robust algorithms or high-intensity activity scenarios. The strong interest in this work and its positive reception in the research community motivated us to follow up with this review, which addresses respiratory monitoring in dynamic and real-world conditions, where motion influence, adaptive filtering, multimodal fusion, and algorithmic robustness become critical.

Recent advances in wearable respiratory sensor technologies are improving non-invasive monitoring. Devices are offered in a variety of forms, including advanced chest belts, adhesive patches, wristbands, and smart textiles, each offering different trade-offs between signal quality, comfort, and robustness [9]. Nevertheless, reliable estimation of RR and V_T_ during complex physical activities remains a major unsolved challenge [16]. Motion artifacts, variability in electrode–skin contact, changes in posture, and non-stationary breathing patterns substantially degrade the quality of the obtained signal. These effects are particularly pronounced in high-intensity scenarios where respiratory signals are strongly modulated by both mechanical motion and neural control mechanisms. While several comprehensive reviews have addressed wearable and remote respiratory monitoring device technologies, fewer studies have critically examined their performance under dynamic real-life conditions with an explicit focus on reliability and robustness [12,17,18]. This gap exists even though the changes in ventilatory parameters during moderate, high-intensity, and prolonged exercise have already been well summarized in the recent literature [2,19].

This paper aims to introduce methodologies for respiratory monitoring by evaluating their hardware implementations and related algorithmic approaches. In addition to individual sensing modalities, this paper also explores the role of multisensor fusion, adaptive signal processing, and machine learning (ML) techniques in improving robustness. The focus is on systems designed for real-life situations. Stationary, bedside, or remotely sensed approaches, such as ballistocardiography mattresses, acoustic sensors, radar, and camera-based systems, are excluded. Preference is given to systems that report quantitative respiratory parameters, in particular RR and, where applicable, V_T_ trends.

In terms of sensing, the approaches evaluated fall into two main categories [12]. The first includes direct respiratory measurement methods that capture physical manifestations, including chest and abdominal motion [20], sensed using resistive [21,22], capacitive [23,24], inductive [25,26], or optical strain sensors [27], bioimpedance measurements [28], and kinematic signals derived from inertial measurement units [29,30,31,32,33] that includes accelerometers, gyroscopes, and magnetometers [34]. The second category covers indirect methods that estimate respiration from modulation effects observed in cardiovascular signals, primarily electrocardiography (ECG) and photoplethysmography (PPG).

The term dynamic real-life conditions refers to ambulatory and physically demanding scenarios ranging from daily activities such as walking and posture transitions to vigorous exercise and competitive sports. Emphasis is placed on monitoring during conditions involving substantial body movement, including running, cycling, fitness activities, and sport-specific applications. This focus is motivated by our ongoing research on multimodal respiratory monitoring in highly dynamic environments, specifically motorsport racing and aerobatic aviation, where large motion artifacts, rapid body movements, vibration, and changing body orientation substantially complicate physiological sensing. Nevertheless, studies involving lower-intensity activities or static posture changes were also included when their findings were relevant for understanding wearable performance under progressively dynamic conditions. Interestingly, several studies have already reported considerable differences in respiratory estimation accuracy between sitting, standing, and walking conditions, suggesting that even relatively subtle biomechanical and physiological changes, such as altered diaphragm mechanics or thoracic stabilization, can significantly influence respiratory sensing performance.

Compared with static respiratory monitoring, dynamic monitoring introduces substantially greater technical and methodological challenges. The primary limitation is the presence of motion artifacts, sensor displacement, unstable skin contact, soft tissue deformation, and activity-dependent changes in respiratory mechanics, all of which can obscure physiologically relevant respiratory components. These problems are especially critical for wearable systems, where unobtrusive long-term use must be balanced against signal quality, sensor stability, energy consumption, and computational complexity. An additional challenge identified throughout the reviewed literature is the considerable heterogeneity of experimental protocols, reference modalities, evaluation metrics, and testing scenarios, which strongly limits direct comparison between studies and complicates objective assessment of technological progress. The need for standardized validation methodologies and benchmarking procedures, therefore, represents an important future direction and is further discussed later in this review.

To ensure methodological transparency, a brief description of the literature search strategy is provided. This review primarily focuses on studies published between 2015 and 2025, while selected earlier seminal works were included when historically or technically relevant. Relevant publications were identified using the ResearchGate, PubMed, Scopus, and Google Scholar databases. The search process combined keywords related to wearable and dynamic respiratory monitoring, including “wearable respiration monitoring”, “respiratory rate estimation”, “tidal volume estimation”, “motion artifacts”, “dynamic conditions”, “exercise monitoring”, “smart textiles”, “bioimpedance”, “seismocardiography”, “ECG-derived respiration”, “PPG-derived respiration”, and “multimodal respiratory sensing”. Emphasis was placed on studies evaluating respiratory monitoring under ambulatory, exercise, or free-living conditions, as well as on recent advances in multimodal sensing, artifact suppression, and machine-learning-based signal processing. To achieve scientific consistency, we define conceptual and statistical frameworks. Validation refers to the assessment of a system’s performance against a gold standard. Reliability denotes the stability of the measurement across different subjects or sessions, encompassing both repeatability (consistency under identical conditions over short intervals) and reproducibility (consistency across different observers, environments, or longer time periods). Robustness is defined as the ability to maintain operational integrity. From a data characterization perspective, breathing pattern recognition involves identifying specific rhythms and timing, often facilitated by respiratory waveform reconstruction. In dynamic conditions, the averaging window is a critical parameter used to stabilize features. Statistically, the strength of the relationship between the wearable and the reference is expressed via linear correlation, quantified by the correlation coefficient. To assess clinical bias and the range of errors, limits of agreement are established through Bland–Altman analysis, while uncertainty is accounted for to quantify the potential dispersion of the measured values. Finally, although metrics such as “accuracy”, “error”, and “agreement” represent distinct statistical entities, we retain the original terminology used in the cited studies to remain faithful to the specific methodologies employed, treating them as collective indicators of performance relative to a reference standard.

The main body of this review is organized according to the above-mentioned sensing paradigms. First, it addresses direct respiratory sensing, followed by derived or modulation-based approaches, and finally hybrid and multisensor systems. Each section has a consistent structure, starting with a brief theoretical background, continuing with representative devices and studies with particular emphasis on sensor design, placement, and signal processing strategies. To maintain readability, individual studies are described briefly, with only the most relevant information included. More detailed experimental results and specific technical parameters are summarized separately in tables provided at the end of each section. Each main section also concludes with a summary that highlights the main findings and limitations of the respective approach. In addition, a separate section is dedicated to relevant review articles to provide a broader context and encourage cross-comparison. This review concludes with a final discussion that synthesizes findings across all methodological groups and outlines the main challenges and priorities for future research.

**Figure 1:**
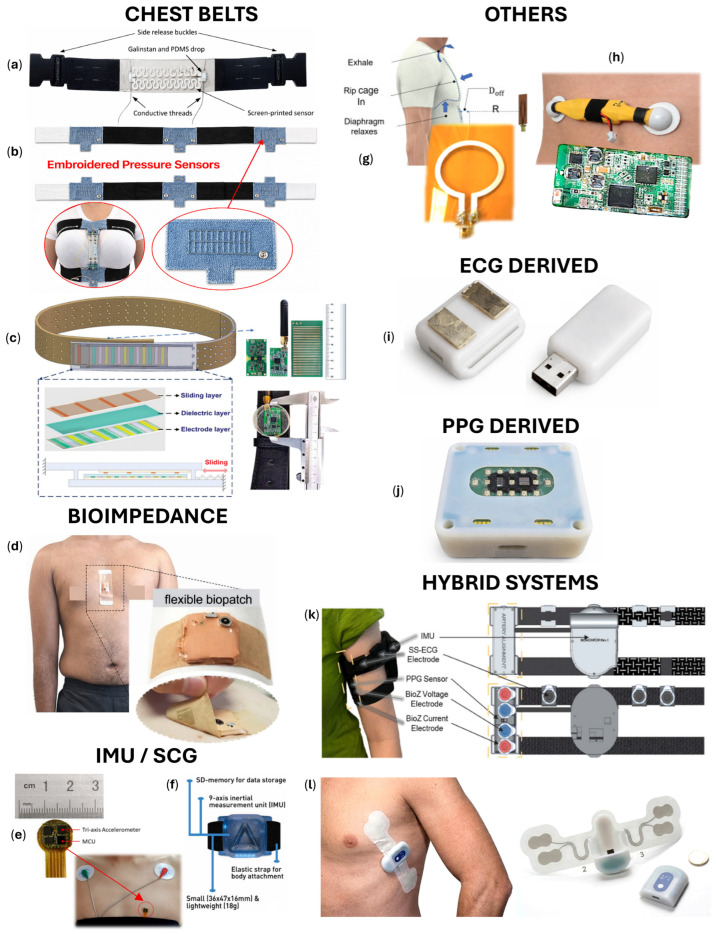
Respiratory monitoring in motion: (**a**) screen-printed resistive chest belt. Reprinted from ref. [35]; (**b**) SolunumWear smart textile system with 6 sensor pads. Reprinted from ref. [36]; (**c**) TENG self-powered chest belt. Reprinted from ref. [37]; (**d**) soft wearable flexible bioimpedance patch using TI ADS1292R. Reprinted from ref. [38]; (**e**) SCG sensor based on 3D accelerometer InvenSense ICM-20602 attached to chest. Reprinted from ref. [39]; (**f**) 9-axis IMU sensor ZurichMOVE. Reprinted from ref. [40]; (**g**) flexible body-integrated antenna system based on near-field coupling printed sensor. Reprinted from ref. [41]; (**h**) EMG-based respiratory device with integrated sound and ECG electrodes. Reprinted from ref. [42]; (**i**) ECG-derived device with minimized electrode distance, TI ADS1292R AFE and ZigBee communications (own design); (**j**) PPG-derived respiratory device with Analog Devices MAX86141 acquisition unit and 15x Osram SFH7016 LEDs (own design); (**k**) multimodal device combining 4-electrode bioimpedance, ECG, PPG, and IMU sensors. Reprinted from ref. [43]; and (**l**) Health Patch—hybrid device combining bioimpedance and ECG with dry electrodes and accelerometric sensor. Reprinted from ref. [44].

An overview of representative wearable respiratory sensors discussed in this work is presented in Figure 1, which serves as a visual reference for the sensing categories addressed in the following sections.

## 2. Direct Measurement Methods

### 2.1. Chest and Abdominal Bands

**Figure 2:**
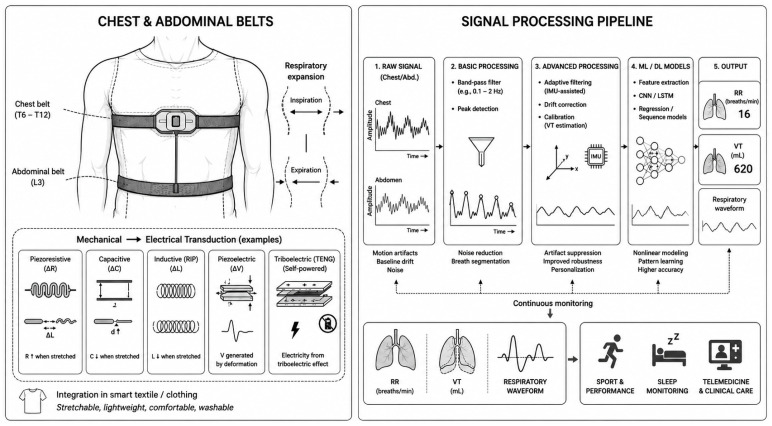
The principle of respiratory monitoring using chest and abdominal belts. The figure was developed through an iterative process combining the author’s manual drawing and generative AI tools, including ChatGPT (GPT-4o; OpenAI, San Francisco, CA, USA) and Gemini (Gemini 3 Flash Image; Google, Mountain View, CA, USA).

Devices based on chest and abdominal motions represent one of the most established and physiologically intuitive approaches (Figure 2) (Table 1). These sensors provide direct access to respiratory mechanics by capturing the cyclic expansion and contraction of the chest and abdomen and converting the resulting mechanical deformation into electrical or optical signals, making them well-suited for ambulatory and activity-related monitoring [13]. Common implementations include elastic strain sensors, inductive plethysmography belts, capacitive transducers, piezoelectric elements, and textile-integrated strain sensors, as well as optically interrogated strain sensors, such as Fiber Bragg gratings (FBGs) [45]. Textile breathing belts and smart clothing represent a significant development as they offer improved comfort, adaptability, and continual wearability while maintaining sensitivity to respiratory movements. Thanks to lightweight and deformable materials, such systems allow unrestricted movement as a necessary condition to use them in real life [19]. To keep the overview structured, the following subsections are organized according to the underlying sensing principle and signal transduction mechanism.

#### 2.1.1. Piezoresistive Systems

Piezoresistive systems represent one of the most widely investigated approaches for wearable respiratory motion sensing due to their high strain sensitivity, simple electronics, flexibility, and compatibility with textile integration. These systems are increasingly incorporated into wearable patches, chest bands, and smart garments for continuous respiratory monitoring. These systems are increasingly integrated into wearable patches or bands due to their high strain sensitivity and compatibility with textile integration.

Chu et al. [46] introduced a crack-based piezoresistive strain sensor fabricated as a thin metal film on a silicone elastomer substrate. The reversible disconnection of microcracks resulted in a high gauge factor, enabling the detection of very small thoracic strains. The system allows simultaneous estimation of the RR and V_T,_ and the results also demonstrated reliable reconstruction of respiratory waveforms. Vanegas et al. [47] designed a system based on a piezoresistive FlexiForce sensor attached to a chest strap system. A compact 3D-printed casing integrated a microcontroller, acquisition circuit, battery, and Bluetooth. Evaluation determined a 27 s analysis window as optimal, yielding low error rates of 4.02% for the time-based algorithm and 3.40% for the counting-based algorithm. In subsequent work, the same group analyzed signal drift induced by movement and body constitution [48]. Loranca Gómez et al. [49] proposed a low-cost piezoresistive textile band and compared its performance with an MPU6050 IMU. While respiratory patterns were reliably detected, the piezoresistive signal exhibited higher susceptibility to noise, suggesting that hybrid sensor fusion with inertial data may enhance robustness for dynamic system applications. Innovations in materials were further demonstrated by Lin et al. [50], who introduced a disposable graphene nanosheet-coated strain sensor, “Motion Tape”, designed for universal integration into elastic chest straps. The modular configuration with a snap fastener allows for interchangeability with existing straps. Validation against a commercial reference demonstrated accurate waveform reconstruction and identical RR measurements. High-intensity validation was described by Di Paco et al. [51], who evaluated a custom chest strap against a metabolic cart during a maximal cardiopulmonary exercise test (CPET) in elite soccer players. The device showed high absolute agreement, a strong linear correlation, and a root mean square error (RMSE) of 2.42 rpm, supporting its applicability even under maximal cardiopulmonary load. Solanki et al. [52] presented a smart-textile system “RespWear”. The system combined a textile pressure sensor belt for monitoring respiration-induced chest motion with a wireless microcontroller-based acquisition unit. Validation was performed on four participants during different breathing rates and body positions, including standing, sitting, bending, and reclining. An OptiTrack infrared camera system served as the reference method. The proposed system achieved a strong correlation coefficient of 0.836 for RR estimation. Later, this system was innovated and named “SolumWear”, with which Cay et al. [36] conducted an evaluation on 10 subjects, confirming the correlation. The system also demonstrated low computational and communication latency, confirming the feasibility of near-real-time monitoring using wearable devices. Screen-printed piezoresistive approaches were introduced by Al-Halhouli et al. [35]. The sensor features a unique silver “horseshoe” pattern electrode on a stretchable substrate to reduce stress concentration and preserve conductivity under high strain. The sensor achieved high RR accuracy across sitting, standing, and Fowler’s 45° position. Egwu et al. [53] proposed a TinyML-based framework for real-time respiratory monitoring using embroidered textile strain sensors. By implementing 8-bit quantized CNN and wavelet-based dense neural network (DNN) architectures on an STM32L4 microcontroller, the system enables fully embedded signal inference directly on the wearable device. Validation on public and custom TexHype datasets demonstrated that the CNN achieved superior accuracy (MAE 1.23 rpm), while the wavelet-based DNN offered lower computational overhead with an incremental power consumption of only 3.3 mW. This study highlights the efficacy of edge AI in balancing the trade-offs between estimation precision, latency, and energy efficiency in smart garment applications.

Sensor placement and multisensor configurations were examined by Laufer et al. [54], who applied regression analysis and bootstrapping techniques to identify optimal thoracic measurement positions for V_T_ estimation. The analysis, using a camera capturing 102 markers, revealed that three circumferential and one distance changes carried the majority of V_T_ information.

Some studies have also increasingly focused on fabrication strategies and textile integration techniques. A study by Tang et al. [55] proposed a smart clothing system based on strain-sensing yarn integrated into fabric using a novel stitching methodology. Using finite element analysis, the authors showed that self-locked yarn configurations and reduced needle pitch improved local stress concentration and enhanced sensing sensitivity. The resulting textile sensor demonstrated a low detection limit of 0.1%, a rapid response time of 280 ms, durability over 10,000 cycles, and strong resistance to washing, humidity, and perspiration. The study also optimized stitch trace lengths for respiration and heartbeat monitoring and integrated the sensor into a complete smart clothing platform. A related fabrication-oriented study by Arslan-Catak et al. [56] developed a sustainable water-based conductive ink composed of carbon nanotubes and cellulose nanocrystals for textile printing. Besides optimizing ink composition, the authors systematically investigated how different textile sensor geometries influence piezoresistive sensitivity during cyclic loading simulating chest movement. Their results demonstrated that sensor geometry significantly affects respiratory sensing performance, highlighting the importance of structural textile design in addition to the conductive material itself.

So far, we have mentioned single-band sensors; now, we will move on to those that measure respiration in multiple channels. Romano et al. [57] integrated two piezoresistive textile sensors, MedTex P130 (Statex Produktions und Vertriebs GmbH, Bremen, Germany), into a smart T-shirt and optimized their size and placement on the chest to improve RR extraction. The system was evaluated on two volunteers against the Zephyr Bioharness 3.0 during sitting, standing, walking, running, and stair climbing. An important finding is that the sensor placed on the side of the chest is more suitable for static activities, the sensor placed on the back for dynamic activities, and the combined output from the sensors outperforms the individual sensors by approximately two times. Di Tocco et al. [58] assessed the feasibility of a multisensor configuration that included four conductive textile strain sensors wired into a Wheatstone bridge to capture thoracic deformation, and combined them with an IMU to estimate HR via mechanical cardiac vibrations. In sitting, standing, and supine positions, and later also on a jockey during horse racing [59], the system achieved consistently low errors in both RR and HR estimation, demonstrating the advantage of distributed sensing for posture robustness.

#### 2.1.2. Piezoelectric Systems

Regarding piezoelectric sensors, Yuan et al. [60] introduced a flexible polyvinylidene fluoride (PVDF) thin-film sensor with a biomimetic lateral line structure inspired by the fish geometry that improved sensitivity to weak thoracic deformations. The device generated stable voltage outputs proportional to V_T_ under varying physiological conditions. Furthermore, Lei et al. [61] investigated a PVDF film encapsulated in polydimethylsiloxane (PDMS) for respiration monitoring during walking conditions. The patch demonstrated robustness against motion artifacts, with RR values showing no significant statistical difference compared to a commercial respiratory effort transducer and maintaining high correlation during ambulation. More application-oriented research was described by Ji et al. [62], who integrated a flexible piezoelectric belt into an aircraft seat system for simultaneous ECG, respiration, and motion monitoring. Extraction of these parameters, combined with a long short-term memory recurrent neural network (LSTM-RNN) classifier, enabled detection of sleep apnea–hypopnea syndrome, with 84–85% accuracy. Although this application was designed for sedentary conditions, the fusion of motion signals with recurrent neural networks illustrates a scalable framework potentially adaptable to dynamic respiration monitoring scenarios. We are leaving the article in the selection because our future research activities will include monitoring respiration during aerobatic flying.

#### 2.1.3. Inductance Systems

Another method, respiratory induction plethysmography (RIP), measures changes in the self-inductance of coils placed around the chest and abdomen. Stretching during inspiration changes the loop inductance, modulating the oscillator frequency and producing a proportional voltage output [63,64]. Accurate estimation of V_T_ requires appropriate weighting of the thoracic and abdominal signals, which is usually achieved by calibration, such as isovolumic or qualitative diagnostic [25]. Ratnagiri et al. [65] analyzed thoracoabdominal motion relationships using the RIP system pneuRIP [66]. An ML elastic-net regularized model identified thoracoabdominal asynchrony, achieving 61.3% accuracy via phase difference and 90.3% using inverse cumulative percentage metrics.

Beyond hardware design, several studies have focused on improving RIP signal processing to enhance respiratory parameter estimation. Holm et al. [67] introduced BreathFinder, an open-source algorithm for RR detection from RIP signals, demonstrating a robust framework for respiratory cycle identification that is also relevant for noise suppression in dynamic conditions. Complementarily, Finnsson et al. [68] proposed a correction and linearization approach for RIP signals against oronasal pneumotachography, substantially improving the accuracy of small respiration and depth estimation. Bias was reduced from 2–12% to 1–9%. Although their study was conducted in the context of sleep pathology, the presented signal correction strategy is highly relevant for wearable monitoring, as it addresses nonlinearities and deformation-related distortions that are likewise encountered during physical activity.

#### 2.1.4. Capacitive Systems

Capacitive stretch sensors estimate respiration by detecting changes in inter-electrode distance during thoracic expansion. Early, conceptually simple implementations convert fabric deformation directly into capacitance changes. Kim et al. [69] validated an easy-wear capacitive belt against a BIOPAC reference, reporting RR errors below 2% across six postures. Enokibori et al. [70] introduced the “Spiro Vest”, an e-textile garment integrating two capacitive length sensors on the chest and abdomen to infer V_T_ from fabric deformation. Vicente [71] evaluated two polyglycerol–sebacate sensor variants—porous and pyramidal microstructures—to estimate tidal volume from capacitance changes. Both variants correlated strongly with a commercial airflow transducer, yielding a mean absolute error (MAE) in the order of 100 mL.

Progressing from these basic textile systems, several studies addressed electrode geometry and materials to improve sensitivity and robustness. Ali et al. [72] proposed a textile sensor that operates without direct skin contact, implemented by screen-printing an electrode stack with an optimized 1:3:1 sensor:reflector:ground ratio on a polyester–cotton substrate. Park et al. [73] developed a flexible PDMS waist belt incorporating silver nanowires and carbon fibers. The design combined enhanced sensitivity and mechanical durability and applied a finite impulse response (FIR) filter to mitigate motion artifacts. Designs prioritizing wearability tackle the trade-off between comfort and measurement fidelity. Kobayashi et al. [74] presented a low-compression smart garment that exerted minimal torso pressure while maintaining strong agreement with spirometry and a mean RR difference below 0.1 rpm across postures.

More complex systems fuse complementary sensing modalities for robust performance in dynamic conditions. Bernhart et al. [75] combined a capacitive pressure interface (PSoC™62) with an elastic belt to detect respiration via pressure changes between the rib cage and the elastic belt, and used an MPU6050 IMU to detect stride and motion. In validation with endurance runners, the multimodal system achieved high F1 scores for step and respiration event detection. At the highest level of complexity are approaches that leverage advanced signal processing, machine learning, and engineered microstructures. Kim and Kim [76] applied a convolutional neural network (CNN) and ResNet architectures to breathing-pattern classification. The optimized ResNet substantially outperformed a conventional CNN (overall accuracy 96% vs. 87%), particularly for the most challenging classes.

#### 2.1.5. Optical Systems

Fiber optic sensors provide a promising alternative to conventional electronic methods. These systems typically operate on the principle of detecting changes in light transmission, such as intensity modulation or macro-bending effects, induced by mechanical expansion of the thorax. A prominent subset of this technology, FBG sensors, is based on wavelength shifts and offers high sensitivity and multiplexing capability, though interrogation cost and temperature cross-sensitivity remain key limitations. For a detailed overview of these sensors and their integration into wearable systems, we recommend the comprehensive review by Krizan et al. [77]. Zha et al. [78] designed a stretchable elastomer optical fiber sensor incorporated into a belt, achieving ≤ 1 rpm RR error and ≤3 bpm HR error, with an MAPE 5.25% and an RMSE of 1.28 rpm during different postures. Huo et al. [79] reviewed the design of flexible optical fiber wearable sensors, highlighting biocompatibility, lightweight design, and integration with IoT and ML for real-time monitoring, while noting challenges in long-term stability and cost that must be addressed to transition these devices into clinical and sports training equipment.

#### 2.1.6. TENG Systems

The growing interest in self-powered sensing has intensified research on triboelectric nanogenerators (TENGs). Li et al. [80] introduced a lightweight retractable sensor based on a rotating thin-film TENG, capable of enduring more than 1 million stretching cycles. When integrated with a Wi-Fi module, an STM32 microcontroller (STMicroelectronics, Geneva, Switzerland), and a charge amplifier, the system generated AC signals proportional to thoracic expansion, enabling wireless respiratory monitoring. Similarly, Shi et al. [37] proposed a compact TENG sensor employing triple-phase interpolation electrodes to quantify thoracic displacement with sub-millimeter resolution and operational durability exceeding 700,000 cycles. Expanding the functional scope of TENG-based monitoring, Xu et al. [81] demonstrated simultaneous measurement of RR and V_T_, achieving an MAE < 0.2 rpm and strong agreement with spirometry. The system also reconstructed volume–time curves with a relative MAE of 2.43%. Beyond triboelectric approaches, Sharma et al. [82] introduced a smart respiration belt utilizing giant magnetoresistance sensing to detect magnetic field variations induced by chest expansion. In a cohort of 12 subjects, the system achieved a maximum deviation of ±2 rpm compared with a BIOPAC reference.

While respiratory masks are often associated with stationary clinical settings, specialized implementations utilizing TENGs warrant inclusion due to their emerging role in high-intensity dynamic environments. This application is particularly relevant to aviation and aerobatics—domains that align with the objectives of our forthcoming research. Zhao et al. [83] demonstrated this potential by integrating an ML-enabled triboelectric textile sensor directly into an oxygen mask for real-time monitoring in extreme conditions. By utilizing plasma-modified surfaces and nanoscale engineering to enhance sensitivity, the system achieved a respiratory pattern recognition accuracy of 97.2%. To ensure reliability during active use, an ML-assisted classifier was employed to effectively distinguish authentic respiratory signals from motion and environmental artifacts.

#### 2.1.7. Commercial Systems

Finally, commercially available wearable respiratory monitoring systems should also be considered, as they represent the practical translation of the previously discussed sensing principles into real-world applications. While most commercial solutions are inherently multimodal, the following section focuses primarily on their dominant respiratory sensing mechanism and its application in dynamic monitoring scenarios.

A representative example is the Airgo belt (MyAir, Inc., Boston, MA, USA) [84], a resistance-based thoracic circumference sensor that integrates stretchable silver-coated yarn and IMU for RR, V_T_, and motion detection. As a CE Class IIa certified medical device, it serves as a non-intrusive proxy for spirometry, respiratory pattern detection, and sleep disorder screening. Validation against a metabolic cart [21] and subsequent analysis using detrended fluctuation analysis [34] demonstrate its reliable performance during both nighttime rest and daytime activity. Notably, this study found that the limits of agreement (LoAs) increased by a factor of approximately three during dynamic activities compared to static conditions [21]. Another widely utilized device is the Zephyr Bioharness 3.0 (Medtronic, Minneapolis, MN, USA) [85]. Because it frequently serves as a reference standard in other studies, its rigorous independent validation is essential. Panni et al. [86] evaluated it against spirometry using Monte Carlo uncertainty propagation, demonstrating high RR accuracy with LoAs within −2 to 3 rpm and a very strong linear correlation. Similar findings were reported by Hailstone and Kilding [87], who confirmed the reliability during dynamic treadmill exercise, and by Kim et al. [88], under more demanding running conditions, including both maximal incremental exercise and prolonged moderate-intensity running in the heat. Their results showed that the Zephyr maintained acceptable agreement with the reference method, although accuracy decreased under more physiologically and environmentally challenging conditions. The device’s test–retest reliability has also been well documented [87,89]. Romano et al. [90] proposed a signal quality index (SQI) algorithm that evaluates the morphology of individual breaths to further enhance the accuracy of such systems. By comparing these waveforms against an average respiratory template, the algorithm successfully identified and excluded unreliable respiratory cycles without the need for an external reference signal. Validation of this SQI across rest, walking, running, and cycling conditions demonstrated consistent improvements in RR estimation accuracy between 2.8% and 30.7%, depending on the situation. Beyond algorithmic enhancements, optimal sensor placement and calibration strategies are critical factors for accurate respiratory monitoring, particularly when estimating V_T_. Investigating these parameters, studies evaluating a thoraco-abdominal sensor (SA9311M, Thought Technology Ltd., Montreal, QC, Canada) demonstrated that thoracic placement (at the T6 and T12 vertebrae) yields more accurate V_T_ estimation than abdominal positioning (at the L3 vertebra) [91,92]. Furthermore, the authors found that implementing an individualized calibration formula significantly outperformed the universal calibration approach. However, it is important to note that during a longitudinal assessment spanning two separate visits, the maximum mean relative error for the V_T_ increased significantly over time, whereas the mean relative error for the RR remained relatively stable.

A notable emerging trend in wearable respiratory monitoring is the miniaturization of traditional full-chest belts into smart patch formats. These patches offer distinct advantages, including reduced size, enhanced user comfort, and the seamless integration of supplementary sensors. Conversely, they typically require direct skin adhesion, and a potential trade-off is a slight reduction in measurement accuracy. This is particularly relevant for V_T_ estimation, as patches cover a significantly smaller surface area of the chest compared to circumferential belts. Exemplifying this technological shift is, for example, the Resmetrix (Resmetrix Medical Ltd., Haifa, Israel) [93], a patch-based, chest-worn system designed for the continuous monitoring of breathing patterns, RR, and V_T_. To support clinical utility, the device transmits data to facilitate the automatic, AI-powered detection of exacerbation-related abnormalities.

Expanding beyond localized patches, another prominent approach involves integrating sensors directly into smart garments and textiles. These systems offer the distinct advantage of capturing data over a larger surface area of the tors, which is highly beneficial for accurate V_T_ estimation. A highly successful commercial solution in this domain is the Hexoskin biometric shirt (Carré Technologies Inc., Montreal, QC, Canada), which combines ECG, respiratory inductance plethysmography (RIP), and 3D accelerometry for multimodal monitoring [94]. Widely adopted in scientific research, its validation against spirometry and 12-lead ECG demonstrated the highest agreement for V_T_ and minute ventilation (VE) during submaximal exercise, though larger deviations were observed at rest and during maximal effort [95]. Heart rate and RR errors remained below 10%, and adjusting for sex and body weight further improved VE estimation. Similarly, Villar et al. [96] reported a minimal RR bias of 0.3 rpm and LoAs of ±2 rpm during submaximal incremental walking. The system also proves effective in natural environments, such as tracking respiration topography in tobacco users, utilizing individualized calibration procedures [97]. Further demonstrating the viability of smart commercial garments under highly dynamic conditions, Innocenti et al. [98] evaluated two vests, the ComfTech vest (Howdy Senior, ComfTech s.r.l.^®^, Monza, Italy) and the Tyme Wear vest (Tyme Wear^TM^, Boston, MA, USA), and the BioHarness 3.0 strap during soccer-specific movements. When compared against a reference metabolic mask, the extracted respiratory frequency closely tracked the reference signal. Breath-by-breath analysis yielded mean absolute percentage errors (MAPEs) of 7.03% for ComfTech, 8.65% for Tyme Wear, and 14.60% for BioHarness. Notably, these errors decreased significantly to 1.85%, 3.27%, and 7.30%, respectively, when the data was averaged over 30 s windows, highlighting the importance of temporal smoothing in dynamic sports applications. What is also visible and can be considered an important conclusion: the vests outperformed the belt system in accuracy.

**Table 1:** Chest- and abdominal belt-based respiratory monitoring.

Sensor Type	Application	Sensing Element	Key Parameters	Ref.
Skin- attached chest strain sensor	RR ^1^, V_T_^2^, respiratory waveform	Crack-based piezoresistive thin metal film	Textile sensor (EeonTex LTT-SLPA-20K), silicone elastomer substrate, miniature, BL ^3^, linear response, MLR ^4^ algorithm for V_T_, validated vs. spirometry, during motion: RR R^2^ = 0.83, V_T_: concordance correlation coefficient 0.75, bias −77 mL, LoAs ^5^ −374–220 mL, SEE ^6^ = 131 mL (26%)	[46]
Chest belt	RR	Piezoresistive sensor (FlexiForce A201)	BLE ^7^, 3D-printed casing integrating microcontroller and acquisition system, 21 subjects, optimal analysis window 27 s, time-based algorithm error 4.02%, counting-based algorithm error 3.40%	[47,48]
Chest belt	RR, respiratory waveform	Disposable graphene nanosheet-coated piezoresistive strain sensor	Snap fastener interface, 4-channel, 12-bit ADC resolution, sampling rate max. 66 Hz, data storage 32 GB mSD ^8^ card, BL, 2000 mAh LiPo battery, 90 min working time, accurate respiratory waveform reconstruction	[50]
Chest belt	RR	Strain-based chest piezoresistive sensor integrated in elastic strap	Validation during maximal CPET ^9^ vs. metabolic cart, BLE, 26 soccer players, high-intensity cardiopulmonary load, high absolute agreement ICC ^10^ = 0.97, linear correlation 0.96, RMSE ^11^ = 2.42 rpm	[51]
Smart textile chest belt	RR	6× embroiled piezoresistive textile pressure sensor	16-bit, sampling rate 64 Hz, BPF ^12^ 0.1–0.35 Hz, wireless data, posture-independent RR estimation, 10 subjects, validated vs. OptiTrack IR ^13^ camera, correlation coefficient 0.836, MAE ^14^: standing (deep 3.25%, normal 12.3%, fast 3.03%), sitting (22.91%, 11.61%, −0.58%), latency: 4.84 s (computational), 2.13 ms (communication)	[36]
Chest belt	RR	Screen-printed piezoresistive sensor	Silver horseshoe-pattern electrode on stretchable substrate, validation vs. airflow, RR evaluated in sitting, standing and Fowler’s 45° position, minimal RR error 0.055 rpm across postures, LoAs −0.91–0.998	[35]
Smart textile	RR	Embroidered meander-pattern textile strain sensor	STM32L4 microcontroller, CNN ^15^ + wavelet-based DNN ^16^, TinyML/embedded edge AI ^17^, public strain-sensor + TexHype dataset, MAE: 1.23 rpm (CNN), 2.21 rpm (DNN), inference latency: 5.8–6.2 s (CNN), 18–96 ms (DNN), power overhead 3.3 mW	[53]
Smart shirt/chest belt	V_T_	3× strain gauges piezoresistive	Optimization of sensor distribution by 102 motion capture points, coefficient of determination 0.97, average V_T_ error 104.4 mL	[54]
Smart shirt	RR	2× piezoresistive textile sensors (MedTex P130)	2 subjects, validated vs. Zephyr Bioharness 3.0, static activities: lateral chest sensor MAE 0.1–0.3 rpm, back sensors MAE 1.1–3.2 rpm, during walking: lateral chest sensor MAE 1.9, back sensor MAE ≈ 0.1 rpm, 9 subjects, during sitting, standing, walking, running, and stair climbing–results by sensor combination MAE to 0.32 rpm, individual sensors: MAE 0.53 rpm and 0.78 rpm	[57]
Two chest belts	RR, HR	4× conductive piezoresistive textile sensors sewn on elastic belts + IMU ^18^	Textile sensor (EeonTex LG-SLPA), IMU (LSM9DS1), µSD storage, 8 h battery life, sampling rate 100 Hz, validated vs. Zephyr BioHarness, 8 subjects, RR average error ~0.17–0.35 rpm (sitting/standing), ~2.95 rpm (supine), RR percentage error ~1.21% (sitting), ~3.49% (standing), ~9.25% (supine)	[58]
Chest sensor	RR, V_T_	Piezoelectric PVDF ^19^ thin-film sensor	Bio-inspired lateral line geometry to enhance sensitivity to low-amplitude thoracic deformation, passive self-powered sensing, stable voltage output proportional to V_T_, BL, low detection limit 0.5 mN, sensitivity 0.24 V/N, response time 4 ms	[60]
Chest patch	RR	Piezoelectric PVDF film encapsulated in PDMS ^20^	Improved mechanical stability, motion-robust during dynamic walking, RR showed no statistically significant difference *p* > 0.05	[61]
Seat belt integrated system	RR, ECG ^21^, motion, OSA ^22^	Flexible piezoelectric belt sensor	Signal fusion with LSTM-RNN ^23^ classifier, ML ^24^-based multimodal framework to respiration analysis with motion context, OSA accuracy 84–85%	[62]
Chest and abdominal belts	RR	2× RIP ^25^ belt	ML-based analysis, regularized model, 51 pediatric subjects, thoracoabdominal asynchrony accuracy: 61.3% (phase difference features), 90.3% (inverse cumulative percentage metric)	[65,66]
Chest belt	RR	RIP	BreathFinder algorithm, 31 subjects, static conditions, dataset comprising 8782 (7.3 h) manually annotated breaths, RR detection accuracy 94%	[67]
Chest belt	RR	Capacitive sensor	Flexible electrodes/dielectric layer, validation vs. BIOPAC MP150, 6 postures, RR MAE < 2% (longer period)	[69]
e-Textile garment	RR, V_T_	2× capacitive textile length sensors	Dual-sensor configuration capturing thoracic and abdominal, BL, 3 subjects, walking, sampling rate 100 Hz, V_T_ error reduction 60%	[70]
Chest attachment	V_T_	Capacitive pressure sensors	Validated vs. airflow, 38 subjects, mean correlation > 0.91, porous substrate: sensitivity 0.09 kPa^−1^, MAE 122 mL, pyramidal substrate: sensitivity 0.015 kPa^−1^, MAE 100 mL	[71]
Textile chest belt	RR	Textile capacitive sensor with screen-printed electrodes	Electrodes on polyester cotton fabric, optimized electrodes ratio 1:3:1 (sensor:reflector:ground), validation vs. manual counting, frequency-based readout, sensitivity 6.2%, RR accuracy 98.68%,	[72]
Waist belt	RR	Capacitive pressure sensor	PDMS dielectric with Ag nanowire and carbon fiber electrodes, optimized for belt, sensitivity 0.161 kPa^−1^, dynamic range 200 kPa, mechanical durability > 6000 cycles, FIR ^26^ filtering to suppress motion	[73]
Smart garment	RR	Double layer capacitive bending angle sensor	Minimizing mechanical constraint, compression pressure 0.77 ± 0.21 kPa, validation vs. spirometry, 20 subjects, strong correlation 0.97–0.99 across postures, mean RR difference < 0.1 rpm	[74]
Chest belt	RR, stride	Capacitive pressure sensor + IMU	Sensing interface (PSoC^TM^62), IMU (MPU6050) for stride and motion detection, validation vs. ergospirometry in endurance runners, F1 score 93.2% (step), 97.4% (exhalation), 97.2% (inhalation)	[75]
Abdominal garment	RR, respiratory waveform	Textile capacitive sensors with embroidery electrodes	100 × 50 mm electrodes, DL ^27^ models, respiratory pattern estimation: accuracy: 0.87 (CNN), 0.96 (ResNet ^28^), precision under challenging breathing: 0.6 (CNN), 0.8 (ResNet)	[76]
Chest belt	RR, HR ^29^	Elastomer optical fiber sensor	10 subjects, different postures, validated vs. manual counting, RR error ≤ 1 rpm, HR error ≤ 3 bpm, MAPE ^30^ 5.25%, RMSE 1.28 rpm	[78]
Chest belt	RR	Flexible optical fiber sensor	Sensor embedded in wearable substrates, compatible with textile integration, enables IoT ^31^ connectivity, different respiratory rates experiment, ML-based real-time monitoring, MAE 0.31 rpm (2.29%)	[79]
Chest belt	RR	Retractable thin-film TENG ^32^ sensor	Self-powered, miniaturized, mechanical durability > 1,000,000 stretching cycles, resolution 0.13 mm, integrated Wi-Fi ^33^ module and STM32 controller	[80]
Chest belt	RR	TENG sensor with triple-phase interpolation electrodes	Self-powered, resolution > 1 mm, mechanical durability > 700,000 stretching cycles, wireless data transmission, compact design suitable for integration into wearable systems	[37]
Chest belt	RR, V_T_	TENG sensor	Self-powered, validation vs. spirometry, MAE < 0.2 rpm for RR, correlation 0.88, V_T_ reconstruction relative MAE 2.43%	[81]
Chest belt	RR	Giant magnetoresistance sensor	Non-contact, integrated into elastic belt, validated vs. BIOPAC, 12 subjects, maximum RR error ± 2 rpm	[82]
Pilot mask	RR, respiratory waveform	Tribolometric fibers in pilot oxygen mask	ML-assisted respiratory pattern classification, sensitivity 2.02 V·kPa^−1^, response time 96 ms, 420% output voltage enhancement, accuracy 97.2%	[83]
Chest belt (commercial)	RR, V_T_, sleep, OSA, activity	Stretchable sensor + IMU	BL, IP67, 6-weeks autonomy, ML and AI analysis, CE Medical Device Class IIa certified, 21 subjects, RR LoA: ±5.9 rpm (standing), ±7.9 rpm (seated), ±10.6 (supine), ±25.8 (low intensity), ±19.5 (medium-high intensity), ±31.5 (maximal intensity), normalized minute ventilation relative median error > 5.9% (standing), 7% (seated), 3.4% (supine), 9.3% (low intensity), 34.7% (medium-high intensity), 40.6% (maximal intensity), α ≈ 0.74–0.75 (RR), α ≈ 0.88–0.97 (V_T_)	[21,34,84]
Chest belt (commercial)	ECG, HR, RR, temp ^34^, activity	ECG electrodes + capacity pressure pad + 3D accelerometer + thermistor	BL, IP55, 12 h battery life, ECG (250 Hz), RR (25 Hz), temp (1 Hz), acceleration (100 Hz), FDA 510(k) CE (Class II), weight 71 g, smoothing and high pass filter, RR accuracy ± 1 rpm, LoAs −2–3 rpm (static and dynamic), ±5 rpm (maximal incremental running test), ±8.3 (running trial in the heat), linear correlation 0.95, typical error 4.4–8.7%, bias −0.6–0.2 rpm, test-retest reliability typical error 1.4–2.8 rpm (4.3–7.3%)	[85,86,87,88]
Chest belt (commercial)	ECG, HR, RR, temp, activity	ECG electrodes + capacity pressure pad + 3D accelerometer + thermistor	SQI ^35^-based approaches, morphology exclusion of unreliable cycles, 33 subjects, MAPE reduction 18.5% (rest), 22.2% (walking), 2.8% (running), 14.1% (cycling), 30.7% (high intensity interval training)	[85,90]
Chest belt (commercial)	RR, V_T,_ respiratory waveform	Stretch-sensitive girth sensor	Requires external DAQ/amplifier, non-calibrated V_T_, individualized calibration, mean relative error 13–26% (RR), 19–35% (V_T_)	[91,92]
Chest patch (commercial)	RR, V_T_, HR, temp, activity	Proprietary stretchable sensor	BL, AI algorithm, respiratory patterns and deterioration	[93]
Smart textile biometric shirt (commercial)	RR, V_T_, ECG, HR, HRV ^36^, sleep, activity, VO_2_^37^ max,	RIP ^16^-based belts + ECG electrodes + 3D accelerometer	RIP (128 Hz); ECG (256 Hz), accelerometer (64 Hz), BL, validation vs. spirometry and 12-lead ECG, 17 subjects, HR and RR errors < 10%, agreement for V_T_ ≤ 5.3% (submaximal exercise), ≤15.3% (rest), ≤11.7% (maximal effort), V_T_ estimation improved with sex and body-weight adjustment (r^2^ = 0.89)	[94,95]
Strain-based systems (commercial)	HR	Strain-based systems	15 soccer players, validated vs. metabolic mask, MAPE: 7.03% (ComfTech), 8.65% (Tyme Wear), 14.60% (BioHarness), LoA: ±12 rpm, ±15.7 rpm, ±24.4 rpm, MAPE (30s averaging window): 1.85%, 3.27%, 7.30%	[98]

^1^ Respiration rate, ^2^ tidal volume, ^3^ Bluetooth, ^4^ multiple linear regression, ^5^ limits of agreement, ^6^ standard error of estimate, ^7^ Bluetooth low energy, ^8^ MicroSD card, ^9^ cardiopulmonary exercise testing, ^10^ intraclass correlation coefficient, ^11^ root mean square error, ^12^ bandpass filter, ^13^ infrared, ^14^ mean absolute error, ^15^ convolutional neural network, ^16^ dense neural network, ^17^ artificial intelligence, ^18^ inertial measurement unit, ^19^ polyvinylidene fluoride, ^20^ polydimethylsiloxane, ^21^ electrocardiography, ^22^ obstructive sleep apnea, ^23^ long short-term memory recurrent neural network, ^24^ machine learning, ^25^ respiratory induction plethysmography, ^26^ finite impulse response, ^27^ deep learning, ^28^ residual network, ^29^ heart rate, ^30^ mean absolute percentage error, ^31^ Internet of Things, ^32^ triboelectric nanogenerator, ^33^ wireless fidelity, ^34^ temperature, ^35^ signal quality index, ^36^ heart rate variability, ^37^ volume of oxygen.

### 2.2. Bioimpedance Methods

**Figure 3:**
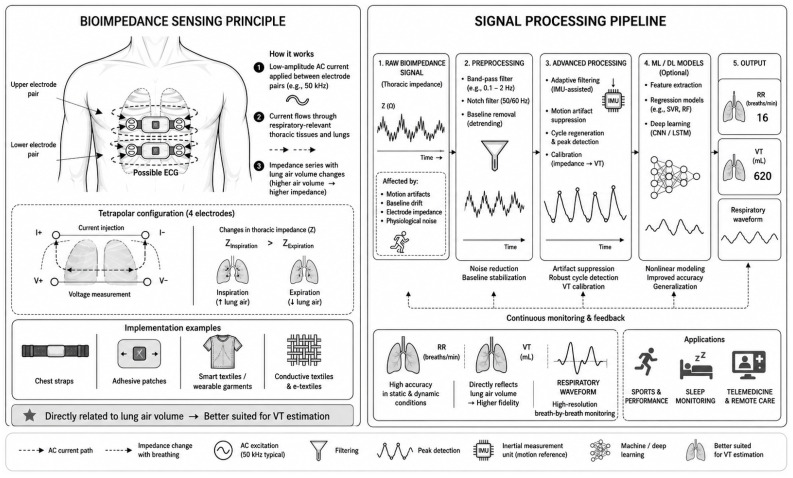
The principle of bioimpedance respiratory monitoring. The figure was developed through an iterative process combining the author’s manual drawing and AI generative tools, including ChatGPT (GPT-4o; OpenAI, San Francisco, CA, USA) and Gemini (Gemini 3 Flash Image; Google, Mountain View, CA, USA).

Bioimpedance-based respiratory monitoring, often also referred to as electrical impedance plethysmography (EIP) (Figure 3) (Table 2), measures cyclic changes in thoracic impedance associated with changes in lung air volume. Since air has a significantly higher electrical resistance than surrounding tissues, inspiration and expiration induce measurable fluctuations that are directly related to RR and V_T_. Bioimpedance is typically measured using surface electrodes arranged in a bipolar or tetrapolar configuration and integrated into chest straps, adhesive patches, or textile platforms. Groenendaal et al. [99] published a comprehensive review of wearable bioimpedance systems. The authors revealed the transition from hospital-based systems to unobtrusive home monitoring. Importantly, this review highlights ongoing challenges, including power consumption and the long-term stability of the electrode–skin interface and motion-induced artifacts.

#### 2.2.1. RR Estimation

Several studies confirmed that bioimpedance-based sensing can provide accurate RR estimation, especially in controlled conditions. The early validation reported by John et al. [100] demonstrated the efficacy of the PhysioPatch against a respiratory chest belt across different breathing rates. Similarly, Heydari et al. [101] evaluated a chest-based system for simultaneous HR and RR monitoring using a TCO_2_ sensor as the reference and reported low RR errors across diverse breathing patterns. Piuzzi et al. [102] further extended this concept by introducing a textile-based thoracic belt that injects a 50 kHz current, enabling simultaneous ECG and respiratory monitoring with high RR accuracy during both quiet breathing and tachypnoea.

More recent developments have focused on patch-based wearable systems suitable for continuous and remote monitoring. Qiu et al. [103] proposed a chest patch capable of accurate RR monitoring across walking, running, and cycling, while also integrating Bluetooth and LoRa communication for telemedicine applications. A similarly designed real-time bioimpedance patch was presented in [35], where RR estimation remained highly accurate in both static and dynamic conditions. In contrast, not all systems retained such performance under physical load. For example, Wei et al. [44] evaluated a “Health Patch” integrating bioimpedance against the metabolic reference system Cosmed K5 [104] and observed only moderate agreement during exercise, highlighting the sensitivity of bioimpedance measurements to motion, contact variability, and physiological perturbations.

#### 2.2.2. V_T_ Estimation

In addition to RR estimation, several studies have focused on V_T_ estimation, where this modality often offers greater physiological relevance than purely motion-based sensing. Early evidence was provided by Berkebile et al. [105], who evaluated a compact multifrequency tetrapolar sternal patch under both static and dynamic conditions against a conventional chest electrode configuration and spirometry. Their results demonstrated a strong Pearson correlation coefficient (0.93 ± 0.05) with reference V_T_ and very low MAPEs of 0.93% for the patch and 0.74% for the chest configuration, even during physical activity, confirming that localized thoracic bioimpedance can capture meaningful ventilatory changes.

A systematic work was presented by Blanco-Almazán et al. [106,107,108], who investigated V_T_ monitoring across multiple perspectives, including electrode configuration, inspiratory loading, and ambulatory monitoring. Across these studies, the authors demonstrated a strong linear relationship between impedance variation and V_T_, high respiration phase detection accuracy in dynamic conditions, and sensitivity to breathing variability during walking. In a complementary mechanistic analysis [109], combining bioimpedance with airflow and accelerometry, they further showed that signal composition changes with inspiratory muscle effort and that, under high inspiratory load, the bioimpedance signal is increasingly influenced by mechanical chest motion. By incorporating both volume and motion-related information into neural-network models, they achieved highly accurate V_T_ estimation (MAPE < 4.29%), indicating that hybrid modeling may be particularly beneficial in dynamic conditions.

More advanced volumetric approaches have moved toward multichannel spatial bioimpedance acquisition. Khan et al. [110] introduced the concept of “virtual spirometry”, using a 10-channel bioimpedance vest and a segregated envelope and carrier (SEC) algorithm, enabling regression-based reconstruction of the respiratory waveform in a form analogous to spirometry. Similarly, Frerichs et al. [111] extended wearable bioimpedance toward electrical impedance tomography (EIT) by integrating 21 replaceable sensors into a textile vest, thereby increasing spatial resolution and the potential to capture regional ventilation patterns.

#### 2.2.3. Algorithm Implementation

Beyond hardware design and electrode placement, the performance of wearable bioimpedance systems depends strongly on the applied signal processing and artifact suppression strategies, so algorithmic robustness has become a key determinant of practical usability in dynamic conditions.

One of the earlier dedicated solutions was proposed by Järvelä et al. [112], who introduced a three-electrode wearable system employing a “dual vector” algorithm to suppress motion artifacts. The signals were processed locally and transmitted wirelessly to a central monitoring station. In a clinical validation, the system achieved a mean RR difference of −0.6 ± 2.5 rpm compared with capnography, demonstrating that even relatively simple algorithmic compensation can substantially improve performance.

As wearable applications moved toward more realistic and less controlled environments, automated signal quality evaluation became increasingly important. Albaba et al. [113] addressed this need by developing a quality classification framework for capacitively coupled bioimpedance signals, designed to distinguish between high-quality and corrupted segments using statistical and spectral features. Their method showed strong robustness, achieving an accuracy of 91% on the primary test set, with sensitivity reaching up to 98%, while a fine Gaussian support vector machine (SVM) classifier achieved balanced accuracy up to 94% using 13 selected features out of 52. A similar trend toward data-driven artifact handling was further demonstrated by Moeyersons et al. [114], who investigated the use of ML methods for separating clean from noisy bioimpedance recordings in 47 chronic obstructive pulmonary disease (COPD) patients. They compared heuristic classification with SVM and CNN approaches, showing that both ML-based methods outperformed the heuristic baseline. Specifically, the SVM achieved an accuracy of 87.77 ± 2.64%, the CNN reached 87.20 ± 2.78%, and both yielded area under the curve (AUC) values above 92.5%, confirming the practical value of learned signal quality assessment for wearable respiratory monitoring.

#### 2.2.4. Non-Chest Sensor Locations

Although chest placement logically provides the highest sensitivity, alternative locations to improve wearability and user comfort of long-term monitoring are also being explored. Goyal et al. [115] evaluated the long-term feasibility of a non-standard thigh-to-thigh placement, focusing on day-to-day variability. In their study, they achieved a high correlation of V_T_ with spirometry, slightly outperforming the thoracic placement. The day-to-day variability in the thighs was also significantly lower compared to the thoracic placement, suggesting improved longitudinal stability despite sensitivity to physiological factors, like food and fluid intake. Sel et al. [116] analyzed signal attenuation by comparing standard thoracic measurements against distal configurations. The study demonstrated a markedly reduced impedance modulation and signal–noise ratio (SNR) at wrist locations but confirmed RR extractability using optimized frequency bands. In a subsequent study [117], gold e-tattoos then enabled wrist-based RR detection. Mathews [118,119] confirmed the same results by systematic evaluation of chest, forearm, wrist-to-wrist, and wrist-to-finger configurations using complex impedance spectroscopy. While thoracic placement yielded the highest modulation of 17% at 64 kHz, distal wrist-to-wrist measurements showed only 0.28% change at 256 kHz. Nevertheless, with appropriate filtering and extraction algorithms, usable respiratory signals were demonstrated, providing a methodological basis for smartwatch-integrated bioimpedance systems.

#### 2.2.5. Integrated Circuits

Recent advances in bioimpedance measurement technology have allowed respiratory monitoring to become a standard feature of modern analogue front-end (AFE) integrated circuits. Typical implementations use low-amplitude AC excitation currents of approximately 8–32 µA at frequencies of 32–64 kHz, allowing for safe and low-noise measurements of thoracic impedance suitable for continuous monitoring by wearable electronics. Among early integrated solutions, the ADS129xR series (Texas Instruments, Dallas, TX, USA) [120] has been widely used. This chip combines 8-channel, 24-bit ECG acquisition with integrated respiration impedance, offering programmable gain, internal references, and flexible sampling for synchronized cardiorespiratory monitoring. Newer devices, such as AFE4960 [121] and AFE4500 [122], provide up to 22-bit bioimpedance resolution, configurable current sources, and targeting the use in compact patch-based and battery-powered designs. Analog Devices also offers comparable integrated solutions. The ADAS1000 (Analog Devices, Wilmington, MA, USA) [123] integrates a 5-channel ECG with dedicated thoracic impedance circuitry, supporting simultaneous ECG and respiration acquisition. For ultra-low-power applications, the MAX30001 [124] incorporates a single-channel bioimpedance function with sub-milliwatt consumption. More recent front-end solutions are specifically optimized for wearable bioimpedance systems. The MAX30002 series [125] provides ultra-low-power single-channel impedance with improved motion tolerance, while the MAX30009 [126] enhances energy efficiency and miniaturization even further. Very promising and used in our last research are multimodal AFEs, such as MAX86178 [127] that integrate bioimpedance, ECG, and PPG within a single chip, reducing board area and enabling synchronized multimodal monitoring. Similarly, AS7058 (ams OSRAM, Munich, Germany) [128] integrates dual PPG and a configurable ECG or bioimpedance, supporting compact multimodal wearable architectures.

Compared to discrete implementations where current sources, amplifiers, demodulation, and ADC stages are implemented separately, fully integrated AFEs significantly decrease system size, power consumption, and complexity. However, they have limited flexibility in choosing the excitation frequency, electrode configuration, and optimization of the dynamic range.

**Table 2:** Bioimpedance-based respiratory monitoring.

Sensor Type	Application	Sensing Element	Key Parameters	Ref.
Chest patch	RR ^1^	BioZ ^2^ system (PhysioPatch)	Different respiratory rates experiment, 10 subjects, validation vs. chest belt, MAPE ^3^ 4.12%, Bland–Altman bias 0.27 ± 0.47 rpm	[100]
Shoulders electrodes	RR, HR ^4^	BioZ electrodes	AFE ^5^ AD5933, sampling rate 500 Hz, BLE ^6^, seated and different respiration speeds, validation vs. TCO_2_ sensor, 10 subjects, RR error < 1 rpm,	[101]
Chest belt	RR, ECG ^7^	Textile BioZ electrodes	BioZ (50 kHz), MSP430 µ-controller, AFE AD8220, CC2500 wireless transceiver, sitting and standing position, resolution 16-bit, 10 subjects, average relative error 1.7%, maximum error 4%, time window 30 s	[102]
Chest patch	RR, temperature	BioZ patch, IMU ^8^, Temp ^9^	AFE AD5933, temperature MLX90632, IMU Bosh BMI160, validation across walking, running and cycling, RR accuracy > 97.8% (static), >98.5% (dynamic), BLE + LoRa ^10^, 150 mAh, 4 h operating time, sampling rate 100 Hz	[103]
Chest patch	RR, HR	Dry BioZ electrodes	Validation vs. Cosmed K5 during exercise, 25 subjects, moderate agreement under physical load with LCCC ^11^ = 0.56, MAE ^12^ 1.2–4.5 rpm	[44]
Sternal chest patch	RR, V_T_^13^	Multifrequency tetrapolar BioZ electrodes	5.1 × 5.1 cm patch, AFE AD5940, patch vs. chest electrode layout, validation vs. spirometer, 14 subjects, V_T_ Pearson correlation coefficient 0.93 ± 0.05 (patch), 0.95 ± 0.05 (chest), RMSE ^14^: 177 mL (patch), 129 mL (chest), RR MAPE from 30 s segment: 0.93% (patch), 0.74% (chest electrode layout)	[105]
Chest BioZ system	V_T_, phase detection, COPD ^15^	BioZ + Accelerometer	Evaluation of electrode placement, ambulatory and dynamic conditions, 10 subjects, strong linearity of BioZ and V_T_ (r > 0.965), MAPE < 11%, phase detection accuracy 96%, neural network combining V_T_ and motion: MAPE < 4.29%	[106,107,108,109]
Chest electrodes	RR, V_T_	BioZ system	10-channel BioZ, AFE AD5933, SEC ^16^ algorithm modeling, 19 subjects, 5 distinct physical activities, SVM ^17^-based regression for reconstruction, dynamic conditions: average RR error 5.81 ± 3.34 rpm (segregated envelope and carrier with wavelet-based)	[110]
Textile vest	EIT ^18^	21× replaceable BioZ electrodes	Wearable EIT, 50 subjects with >125,000 EIT images, good-to-excellent ventilation imaging in 34 participants	[111]
3× chest electrodes	RR, tachypnea	BioZ system	Local dual-vector preprocessing to suppress motion, wireless transmission, validation vs. capnography, 40 subjects, mean RR difference −0.6 ± 2.5 rpm	[112]
Capacitively coupled chest electrodes	Respiratory waveform	BioZ electrodes	Quality classification framework distinguishing high-quality vs corrupted segments, statistical and spectral feature extraction, accuracy 91%, sensitivity 98%, balanced accuracy 94%, fine Gaussian SVM with 13 out of 52 selected features	[113]
Chest BioZ system	Artifact detection in respiratory signals	BioZ device (ROBIN imec)	Separation of clean vs. noisy signals, heuristic, SVM, and CNN ^19^ approaches, validation vs. TSD107 Biopac, 47 subjects, accuracy: 84.69 ± 2.32% (heuristic), 87.77 ± 2.64% (SVM), 87.20 ± 2.78% (CNN), AUC ^20^ > 92.5% (SVM, CNN)	[114]
Thigh-to-thigh system	RR, V_T_	Dry BioZ electrodes on the seat	Non-standard placement for improved comfort, AFE MAX30001, 80 kHz signal, Validation vs. spirometry, 5 subjects, V_T_ correlation: 0.94 ± 0.03 (thighs), 0.92 ± 0.07 (chest), Day-to-day variability: 14% (thighs), 40% (chest)	[115]
Distal BioZ sensors/e-tattoos	RR	BioZ electrodes, 35 × 5 mm gold e-tattoos	Distal vs. thoracic placement, reduced BioZ modulation and SNR at wrist, RR using optimized frequency bands, RMSE < 13% and MAE 0.3% for wrist-based e-tattoo	[116,117]
Body electrodes	RR	Thoracic and distal BioZ electrodes	Electrodes configurations: chest, forearm, wrist-to-wrist, wrist-to-finger, TI AFE4300 and MAX30009, complex BioZ spectroscopy: 64–256 kHz, thoracic placement modulation 17% at 64 kHz, wrist-to-wrist 0.28% at 256 kHz, filtering enables detection even in low SNR ^21^	[118,119]
Integrated circuit	Respiration, ECG, EEG ^22^	BioZ AFE ADS129xR	8-channels, 24-bit AFE, sampling rate 250 Hz–32 kHz, −115 dB CMRR ^23^, internal oscillator	[120]
Integrated circuit	Respiration, ECG	BioZ AFE AFE4960	2-channels, 22-bit, single ADC ^24^, SPI ^25^ and IC ^26^ interface, sine wave or square wave excitation	[121]
Integrated circuit	Respiration, ECG, HR	BioZ AFE AFE4500	4-channel, 22-bit, single ADC, SPI and IC interface	[122]
Integrated circuit	Respiration, ECG	BioZ AFE ADAS1000	5-channels and one driven lead, serial interface SPI/QSPI ^27^, AC ^28^ and DC ^29^ lead-off detection	[123]
Integrated circuit	Respiration, ECG	BioZ AFE MAX30001	High input impedance (>1 GΩ), High-Speed SPI interface, 32-Word ECG and 8-Word BioZ FIFOs ^30^, EMI ^31^ filtering, ESD ^32^ protection, DC leads-off detection	[124]
Integrated circuit	Respiration	BioZ AFE MAX30002	Ultra-low-power 158 mW at 1.1 V, 20-bit ADC, 17-bit effective resolution, sampling rate 25–64 Hz, SPI interface	[125]
Integrated circuit	Respiration	BioZ AFE MAX30009	2 and 4 electrode configurations, ultra-low power 250 mW at 1.8 V, 20-bit ADC, 17 bits effective resolution, sampling rate 16 Hz–4 kHz, SPI and IC interface	[126]
Integrated circuit	PPG ^33^, ECG, respiration	BioZ AFE MAX86178	PPG up to 6× LEDs and 4 photodiodes, 8-bit LED drivers, 20-bit ADC, ECG (0.05–40 Hz), low-noise 17-bits, Stimulus 16 Hz–500 kHz, ultra-low power, 115 dB SNR	[127]
Integrated circuit	PPG, ECG, respiration, EDA ^34^	BioZ AFE AS7058	2× ADC (20-bit) for PPG acquisition, 1× ADC (20-bit) for ECG/BioZ acquisition, SPI and IC interface	[128]

^1^ Respiration rate, ^2^ bioimpedance, ^3^ mean absolute percentage error, ^4^ heart rate, ^5^ analog front-end, ^6^ Bluetooth low energy, ^7^ electrocardiography, ^8^ inertial measurement unit, ^9^ temperature, ^10^ long Range, ^11^ Lin’s concordance correlation coefficient, ^12^ mean absolute error, ^13^ tidal volume, ^14^ root mean square error, ^15^ chronic obstructive pulmonary disease, ^16^ segregated envelope and carrier, ^17^ support vector machine, ^18^ electrical impedance tomography, ^19^ convolutional neural network, ^20^ area under the curve, ^21^ signal-to-noise ratio, ^22^ electroencephalography, ^23^ common-mode rejection ratio, ^24^ analog-to-digital converter, ^25^ serial peripheral interface, ^26^ inter-integrated circuit, ^27^ quad serial peripheral interface, ^28^ alternating current, ^29^ direct current, ^30^ first-in, first-out, ^31^ electromagnetic interference, ^32^ electrostatic discharge, ^33^ photoplethysmography, ^34^ electrodermal activity.

### 2.3. Inertial Measurement Units and Seismocardiography

**Figure 4:**
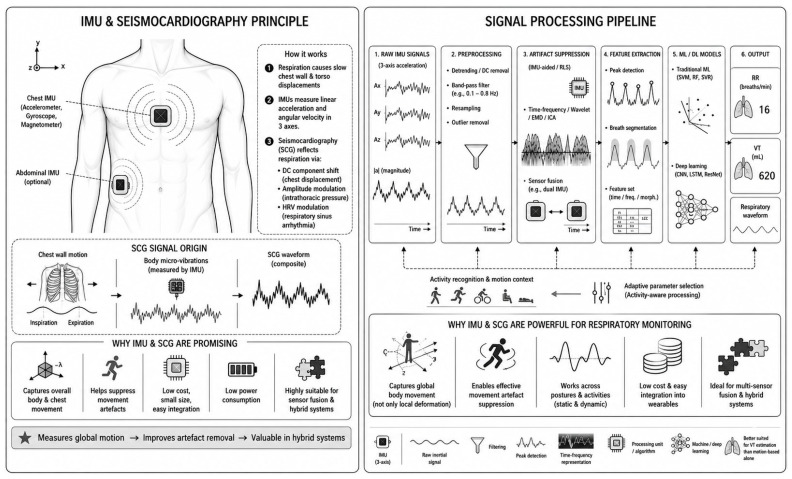
The principle of IMU and SCG respiratory monitoring. The figure was developed through an iterative process combining the author’s manual drawing and generative AI tools, including ChatGPT (GPT-4o; OpenAI, San Francisco, CA, USA) and Gemini (Gemini 3 Flash Image; Google, Mountain View, CA, USA).

Inertial measurement units (IMUs) (Figure 4) (Table 3), which include accelerometers, gyroscopes, and magnetometers, are widely used in wearable devices due to their easy integration [129]. Respiratory activity in inertial signals is primarily manifested as slow, quasi-periodic displacements of the chest wall, which can be separated from faster motion components using bandpass filters, wavelet transforms, empirical mode decomposition, or independent component analysis [130,131]. The scientific discipline that uses these processes is called seismocardiography (SCG) [132]. Respiration affects the measured waveform through several mechanisms: displacements of the DC component caused by chest movement, amplitude modulation related to changes in intrathoracic pressure, and indirectly by modulation via respiratory sinus arrhythmia, which manifests itself in heart rate variability (HRV) [133]. Separating respiratory components starts to become difficult under dynamic conditions, where gross body motion often exceeds the amplitude of respiratory-induced micromotions. To address this problem, recent studies have proposed adaptive and activity-aware processing techniques, including recursive least-squares filtering [40], time–frequency distribution analysis [134], and quantitative modeling of motion artifacts [135]. Sensor fusion strategies, such as using two cooperating accelerometers placed at different locations, can also improve robustness [136].

#### 2.3.1. Hardware-Oriented Research

An excellent starting point for analyzing the current state of hardware-oriented research is the early work of Tadi et al. [137]. The study demonstrated the feasibility of extracting both cardiac and respiratory activity from SCG using an MMA8451Q (Freescale Semiconductor, Austin, TX, USA) accelerometer. A major strength is its rigorous validation strategy, which correlated SCG-derived parameters not only with ECG and chest belts, but also with computed tomography (CT) imaging to link signal morphology to anatomical displacement of the heart. The results confirmed that accelerometer-based SCG can provide highly accurate information on HR and RR variability. Further progress in SCG hardware was presented by Andreozzi et al. [138], who introduced a dome-shaped force-sensing resistor (FSR03CE, Ohmite Mfg Co., Warrenville, IL, USA) for SCG acquisition. Validated against ECG-derived respiration and resistive bands, the system proved to be suitable for real-time biofeedback applications. Despite its hardware simplicity and user-oriented design, the device demonstrated reliable respiratory tracking.

Several studies have also explored the broader use of inertial sensing for unobtrusive respiratory monitoring. Valdés Tirado et al. [139] presented a custom-designed wearable IMU optimized for cardiorespiratory monitoring, detailing hardware characterization and parameter tuning required for high-precision sensing in sports and rehabilitation contexts. Similarly, Ikarashi et al. [140] addressed the limitations of conventional respiration measurement techniques, such as thermistor and impedance methods, by investigating clothing-attached, non-contact sensing with a 6-axis IMU. Frequency-domain analysis confirmed accurate RR estimation even without direct skin contact, supporting the feasibility of low-burden monitoring in everyday environments.

A more integrated wearable concept was proposed by Rahman et al. [141] through the “CardioResp Device”, which integrated inkjet-printed ECG electrodes with a 6-axis IMU. Robustness across static and dynamic postures was achieved using a quaternion-based update algorithm together with multi-stage filtering. Validation against a Vernier Go Direct chest belt demonstrated overall accuracies of 99.3% in static and 98.6% in dynamic conditions.

Significant contributions to IMU-based dynamic monitoring were made by the research group led by Angelucci. Their Wireless Body Sensor Network (WBSN) [29] utilized three chest-mounted IMUs for RR detection and a wrist-worn unit with a PPG sensor for HR monitoring. In the wrist unit, RR is derived directly using the embedded algorithm of the MAX32664C sensor. Data from all units are transmitted via ANT protocol to a smartphone for storage and analysis. The system achieved an RMSE of 3.77 rpm during cycling. In their most recent study [30], they extended the architecture to simultaneous RR estimation and human activity recognition using 9-axis quaternion computation on an nRF52832 microcontroller, implementing the Madgwick gradient descent algorithm [142]. The sensor network consisted of three IMUs: two placed on the chest wall (thorax and abdomen), and a third positioned on the lower back. Their subsequent work [143] introduced a dual-IMU chest–back differential configuration for RR and V_T_ estimation.

#### 2.3.2. Software-Oriented Research

Beyond hardware innovations, a substantial portion of recent studies has focused on improving algorithmic evaluation. Early algorithmic approaches primarily relied on frequency-domain and morphology-based analysis. For example, Pandia et al. [144] performed a detailed spectral investigation of SCG signals acquired using a MEMS LIS3L02AL accelerometer (STMicroelectronics, Geneva, Switzerland). By systematically analyzing the 0–100 Hz band and dividing it into 5 and 10 Hz sub-bands, they identified significant respiratory-related spectral differences in the 10–40 Hz range, thereby establishing a basis for frequency-selective feature extraction. Along similar lines, Dhar et al. [145] investigated SCG morphological changes induced by respiration and exercise, further supporting the physiological sensitivity of SCG to respiratory modulation.

Building on such signal-level observations, several studies introduced increasingly structured feature extraction and ML frameworks. Sadat-Mohammadi et al. [146] combined low-cost accelerometry with 4 different ML approaches to identify physical demand from the respiratory pattern. Likewise, Sandler et al. [147] employed supervised SVM classification for inspiratory/expiratory phase detection. Their methodology was based on ECG-guided segmentation of SCG events and feature extraction from median waveform amplitudes within contiguous 4 ms windows. In a more computationally efficient direction, Ku et al. [148] developed an RR estimation algorithm combining Gaussian averaging filtering with a complex Morlet wavelet scalogram. Bhongade et al. [149] proposed “ResPara-Net,” a system combining a single IMU with a deep convolutional neural network during daily activities. The model achieved low RMSE values of 0.14, 0.12, and 0.13 for normal, fast, and slow breathing, respectively, while normalized MAE remained below 4% across all subjects. Correlation coefficients ranged from 64.47% to 71.53%.

As the field progressed, attention increasingly shifted toward more adaptive and data-driven architectures capable of handling dynamic and noisy real-world conditions. Steinmetzer and Michel [150] proposed a 1D convolutional recurrent neural network (1D-CRNN) trained on IMU data acquired from a smart e-textile. By combining convolutional layers for local feature extraction with recurrent layers for temporal context modeling, the architecture enabled robust segmentation of breathing-related activity from noise. Similarly, Hung et al. [151] introduced an alternative waistband configuration using dual IMUs together with a ResNet-based deep learning (DL) model. Their system effectively separated respiration from stride-induced motion artifacts during running. In a related effort targeting volumetric estimation, Ba et al. [152] employed the Xsens DOT sensor platform and developed a DL framework for V_T_ estimation, integrating a nonlinear high-gain observer with a CNN-LSTM network. Their approach demonstrated substantial robustness even under repeated sensor removal and re-wearing.

Another important research direction has focused on improving robustness through signal decomposition, posture-aware modeling, and adaptive signal quality handling. Azad et al. [153] examined postural and longitudinal SCG variability over a five-month period, showing that while SCG patterns remain relatively stable over time, they are strongly dependent on posture. To mitigate this influence, the authors applied unsupervised ML to group signals into two clusters with reduced waveform heterogeneity. Shipper et al. [154] addressed the problem from a complementary perspective by combining recursive and constrained principal component analysis (PCA) with an SQI for RR estimation, achieving LoAs below 1.45 rpm with at least 80% temporal coverage across variable postures. Similarly, Cheng et al. [155] optimized a dual-IMU chest/back configuration using a combination of PCA, discrete Fourier transform (DFT), empirical mode decomposition, Savitzky–Golay filtering, and Butterworth bandpass filtering. Validation using Xsens DOT sensors [156] against a TI ADS1298R reference demonstrated an RMSE < 0.8 rpm and a correlation coefficient > 0.7 across dynamic scenarios, including standing, sitting, walking, and squatting.

#### 2.3.3. Sensor Location Optimization

Sensor placement critically influences SCG morphology, and therefore, a lot of research has been devoted to optimizing sensor position and orientation. Romano et al. [157] investigated optimal locations for skin-interfaced IMU sensors by collecting accelerometric data from subjects during rest and walking. Their results identified the mitral valve level as the most promising placement, with the dorsoventral axis providing the most informative signal for respiration monitoring. In a related study, the same authors also provided a comprehensive comparison of chest-worn accelerometers and gyroscopes for simultaneous HR and RR monitoring [158]. By evaluating both sensing modalities across multiple positions and analysis window lengths, they showed that accelerometers consistently outperformed gyroscopes in estimating both HR and RR, while improvements became marginal beyond 25 s analysis windows.

Further evidence for the importance of sensor localization was provided by Demirsoy et al. [159], who quantified SCG variability across 16 torso locations. Their findings highlighted the need to minimize sensor drift and to account for axis-specific variability when developing generalized monitoring models. Similarly, Utama et al. [160] investigated optimal gyro-accelerometer placement and reported their best performance when the sensors were positioned on the stomach and chest, with the highest recorded error remaining as low as 2.06%. A comparable multi-location validation was performed by Centracchio et al. [161], who simultaneously evaluated 16 accelerometer positions in nine subjects using a respiratory belt as the reference.

**Table 3:** IMU/SCG-based respiratory monitoring.

Sensor Type	Application	Sensing Element	Key Parameters	Ref.
Chest IMU ^1^	RR ^2^, HR ^3^	3D MEMS ^4^ accelerometer (MMA8451Q)	Sampling rate up to 800 Hz, validation vs. ECG ^5^, chest belt, and CT ^6^ imaging, Pearson correlation coefficient 0.995, 0.998, and 0.994, standard deviation 1.7, 1.8, 8.9 rpm ^7^ for normal (11.1 rpm), slow (6.7 rpm), and fast breathing (23.3 rpm)	[137]
Body attachment SCG ^8^	RR	Dome-shaped force-sensing resistor (FSR03CE)	Validated vs. EDR ^9^ and chest belt, 7 subjects, NI-USB6009 DAQ board, 13-bit, sampling rate 5 kHz, RR accuracy 0.98 (slope 0.99, intercept 0.026 s), LoAs ^10^ ± 0.61 s, respiratory acts detection sensitivity 100%, PPV ^11^ 98.9%	[138]
Custom wearable IMU	RR, HR	6-axis IMU (LSM6DSL)	Sampling rate up to 4 kHz, range ± 4 g, ±250 dps ^12^, processing cycle 220 µs, power consumption 8.5 mA, error characterized via Allan deviation and PSD ^13^	[139]
Clothing attached	RR	6-axis IMU (MPU-6050)	Validated vs. BioZ ^14^, 5 subjects, sampling rate 100 Hz, non-contact measurement, frequency-domain analysis	[140]
Integrated patch (CardioResp)	RR, ECG	6-axis IMU + Inkjet-printed ECG electrodes	Validated vs. Vernier Go Direct chest belt, 10 subjects, BLE ^15^, quaternion-based update algorithm, multi-stage filtering, accuracy 99.3% (static), 99.2% (walking), 98% (running), 98.6% (cycling), MAE ^16^ 0.13 rpm (static), 0.17 rpm (walking), 0.36 rpm (running), 0.23 (cycling)	[141]
Chest and wrist IMUs	RR, HR	3× IMU (ICM-20948) + PPG ^17^ (MAXM86161)	Wireless Body Sensor Network, ANT ^18^ protocol transmission, IMU (10 Hz), HR (1 Hz), embedded HR algorithm 30 subjects, RR RMSE ^19^ 3.77 rpm (cycling)	[29]
Thorax, abdomen, lower back sensors	RR, HAR ^20^	3× 9-axis IMU	Wearable sensor network, nRF52832 µprocessor, sampling rate 40 Hz, 20 subjects, Madgwick gradient descent algorithm, ANT protocol, AI ^21^ method: accuracy of HAR 97%	[30]
Dual-IMU wearable band	RR, V_T_^22^	2× IMU (MPU-6050)	Dual-IMU chest–back differential configuration, SAMD21G18A microcontroller, IC ^23^, BLE, 15 mAh battery, RR correlation r = 0.92, mean difference −0.27 rpm, LoAs +1.16/−1.75 rpm, RR MAE 1.15%, V_T_ MAE < 5%.	[143]
SCG patch	RR	MEMS accelerometer (LIS3L02AL)	18 subjects, frequency-domain analysis of inspiration, expiration, and apnea, significant spectral differences identified in the 10–40 Hz range	[144]
Chest belt	Respiratory waveform	Accelerometer + RIP ^24^	Sample rate 1 kHz, resolution 16-bit, 15 subjects, during physically demanding tasks, different ML ^25^ algorithm for physical demand classification: mean accuracy 90.5% (SVM ^26^), 91.3% (KNN ^27^), 93.4% (RF ^28^), 90.2% (ANN ^29^)	[146]
Chest belt	RR, phase detection	SCG	Respiratory phase detection, 15 subjects, validated vs spirometry, SVM model, accuracy 90.2 ± 6.5%	[147]
Datasets	RR	SCG and PPG	CEBS ^30^ (PhysioNet) datasets, paced and spontaneous respiration, 20 subjects, STMicroelectronics LIS344ALH IMU, complex Morlet wavelet scalogram, Gaussian averaging filter, validated vs. magnetic field-based sensor during 15 activities, 16 subjects, LoAs 95%	[148]
Chest worn	RR, Respiratory waveform	IMU	ResPara-Net DCNN ^31^ algorithm, RMSE: 0.14 rpm (normal), 0.12 rpm (fast), 0.13 (slow breathing), correlation coefficient 64.47–71.53%, MAE: <4%	[149]
Smart e-textile	RR	2× IMU (Adafruit BNO085)	Abdomen and spine IMU, sampling rate 330 Hz, 1D-CRNN ^32^ architecture, 59 subjects, 2000-sample window, mean accuracy 0.88, F1-score 0.92, best case accuracy 99.5%, near-real-time processing	[150]
Dual IMU waistband	RR	2× IMU	ResNet-based DL ^33^ model, 20 subjects, sampling rate 10 Hz, 32 s windows, separation of respiration from stride-induced motion artifacts, outperformed PCA ^34^ and relative angle baselines during running, MAPE 9.1% (sit), 8.9% (stand), 20% (walk), 9.9% (run)	[151]
Chest and abdominal IMUs	V_T_	4× IMU (Xsens DOT)	High-gain observer combined with CNN ^35^-LSTM ^36^, 6 subjects, averaged RMSE 40.38 mL, robust to sensor drift and repeated re-wearing	[152]
Chest worn	RR	Accelerometer (ADXL355)	Sampling rate 250 Hz, recursive and constrained PCA, signal quality index, 20 subjects, variable postures, LoA < 1.45 rpm, ≥80% temporal coverage,	[154]
Chest/back IMUs	RR	2× IMU (Xsens DOT) on chest/back	PCA, DFT ^37^, empirical mode decomposition, Savitzky–Golay + Butterworth filtering, validated vs. TI ADS1298R (dynamic scenarios), RMSE < 0.8 rpm, correlation coefficient > 0.7	[155]
Body attached IMU	RR, HR	IMU (Xsens DOT)	Sampling rate 120 Hz, 15 subjects (rest and walking), optimal location—mitral valve level, most informative-dorsoventral axis, HR MAE < 1.5 bpm, RR MAE < 4 rpm, accelerometer outperformed gyroscope in accuracy, diminishing returns beyond 25 s analysis windows	[157,158]
16× SCG on torso	Respiratory phase	Accelerometers (ADXL355)	Sampling rate 500 Hz, evaluated across 16 torso locations, accuracy 92% (location), 90% (respiratory phase)	[159]
Body attached	RR	Gyroscope + Accelerometer	GY-521 MPU6050 IMU, paced breathing, highest error 2.06% at 25 rpm	[160]
Multi-accelerometer setup	Respiratory	Accelerometers (ADXL355)	Sampling rate 500 Hz, 16 simultaneous body positions, 9 subjects, validated vs. chest belt, average sensitivity and PPV 95.8%/95.5% (chest inclination), 85.9%/84.4% (AM ^38^), 94.3%/95.7% (morphological similarity index)	[161]

^1^ Inertial measurement unit, ^2^ respiration rate, ^3^ heart rate, ^4^ microelectromechanical systems, ^5^ electrocardiography, ^6^ computed tomography, ^7^ respirations per minute, ^8^ seismocardiography, ^9^ ECG-derived respiration, ^10^ limits of agreement, ^11^ positive predictive value, ^12^ degrees per second, ^13^ power spectral density, ^14^ bioimpedance, ^15^ Bluetooth low energy, ^16^ mean absolute error, ^17^ photoplethysmography, ^18^ advanced and adaptive network technology, ^19^ root mean square error, ^20^ human activity recognition, ^21^ artificial intelligence, ^22^ tidal volume, ^23^ inter-integrated circuit, ^24^ respiratory induction plethysmography, ^25^ machine learning, ^26^ support vector machine, ^27^ k-nearest neighbors, ^28^ random forest, ^29^ artificial neural network, ^30^ combined measurement of ECG, breathing and seismocardiograms dataset, ^31^ dense neural network, ^32^ one-dimensional convolutional recurrent neural network, ^33^ deep learning, ^34^ principal component analysis, ^35^ convolutional neural network, ^36^ long short-term memory, ^37^ discrete Fourier transform, ^38^ amplitude modulation.

### 2.4. Other Methods

Beyond chest belts, bioimpedance, and IMU sensing, several alternative sensing principles have also been explored, including pressure-based airflow sensing, electromyography (EMG), acoustic analysis, and radiofrequency resonant methods (Table 4).

Massaroni et al. [162] developed a device measuring breath-induced pressure drops at the nostril level during physical exercise. The system demonstrated high robustness under dynamic conditions. This approach offers direct quantification of an airflow while maintaining compatibility with dynamic scenarios, although nasal interfaces may limit long-term comfort compared to completely unobtrusive modalities.

Surface EMG has also become one of the surrogate markers of respiratory effort. Gronska et al. [163] evaluated diaphragmatic EMG features across time, frequency, and statistical domains. Correlation with gold-standard esophageal pressure measurements showed that time-domain features, particularly filtered envelope, RMS, and waveform length, achieved moderately strong correlations with respiratory effort. Notably, waveform length and slope sign change remained robust even in low-quality signals, highlighting the resilience of selected EMG-derived methods. Extending this concept, George et al. [42] proposed a multimodal configuration combining diaphragmatic and intercostal EMG with a piezoelectric microphone. This dual-sensor configuration enables a more comprehensive assessment of respiratory mechanics and sounds, offering a robust solution. In EMG-based respiratory monitoring, advanced DL frameworks are increasingly utilized for robust signal reconstruction and modality mapping. Huang et al. [164] developed a cascaded CNN–LSTM architecture for diaphragm EMG that effectively suppresses ECG interference and quantifies nonlinear motion. This model achieved a Pearson correlation of 0.95 ± 0.03 without requiring additional post-processing. Addressing the complex mapping between different physiological sequences, Chen et al. [165] introduced a Multi-Scale Patch Transformer. By incorporating an Attention-based modality transition module for cross-sequence EMG-to-respiration forecasting, this framework outperformed conventional state-of-the-art models.

As discussed above, acoustic sensing may be another way forward. Liu et al. [166] presented the “EarMeter”, which is an in-ear system embedded in conventional earbuds for V_T_ estimation from internally propagated breathing sounds. To address weak acoustic coupling and motion interference, the authors implemented a deep learning framework using transfer learning from high-quality nasal sound and physiological cardiorespiratory coupling. In LOSO validation, the system demonstrates the feasibility of continuous monitoring at the consumer level. Clinical-grade acoustic validation was provided by Abdulsadig et al. [167] in their assessment of the AcuPebble RE100 (Acurable, London, UK). Compared with capnography and polygraphy, the wearable sensor meets performance levels compatible with medical device standards. Together, these studies indicate that on-body acoustic monitoring supported by robust algorithms can achieve clinically meaningful RR accuracy.

Non-contact and textile-integrated electromagnetic approaches represent an alternative. Abounasr et al. [41] proposed an electromagnetic coupling system based on a loop antenna and a flexible split-ring resonator tag. The 50 × 50 mm conformal sensor, fabricated by inkjet and extrusion printing on polyimide and polyethylene terephthalate (PET) substrates, detects chest wall displacement via resonant frequency shifts, achieving a sensitivity of 1.7 MHz/mm and strong correlation with the BIOPAC respiratory belt. Similarly, Gharbi et al. [168] developed a textile-integrated embroidered loop antenna embedded in an abdominal belt, coupled with a compact Bluetooth transmitter. Respiratory motion modulates antenna resonance through mechanical stretching, enabling wireless respiration tracking within garment-integrated architectures.

**Table 4:** Other methods for respiratory monitoring.

Sensor Type	Application	Sensing Element	Key Parameters	Ref.
Nasal device	RR ^1^, V_T_^2^	Pressure sensor (SDP610, Sensirion)	Breath-induced pressure drops at nostril level, evaluated during physical exercise, RR percentage error 4.03%, 30 s window averaging error 2.38%, HIIT ^3^ test LoA ^4^ ±1.6 rpm	[162]
Surface EMG ^5^	Respiratory effort, OSA ^6^	EMG electrodes	Evaluated diaphragmatic EMG features, validated vs. esophageal pressure, 10 subjects, time-domain (filtered envelope, RMS ^7^, waveform length), moderately strong correlation R > 0.6, robust in low-quality signals R > 0.5	[163]
Surface EMG + acoustic	RR, V_T_, sounds	EMG electrodes + piezoelectric microphone	Combined diaphragmatic and intercostal EMG with microphone, 2 subjects, mean AUC ^8^ 0.4–1.23 × 10^8^ for V_T_ (500–1000 mL)	[42]
Surface EMG	Respiratory waveform	EMG electrodes on diaphragm	CNN ^9^–LSTM ^10^ + multi-scale CNN, 49 subjects, 0.95 ± 0.03 correlation coefficient, ECG ^11^ artifact suppression without post-processing, real-time monitoring	[164]
In-ear acoustic system	V_T_	Microphone	DL ^12^ framework with transfer learning, internally propagated breathing sounds, LOSO ^13^ validation, validated vs. VO_2_Master, average MAPE ^14^ 18.19%	[166]
Acoustic sensor	RR	Acoustic sensor AcuPebble RE100	Validated vs. capnography and polygraphy, RMS deviation < 3 rpm, MAE ^15^ 1.83 rpm ^16^	[167]
Flexible electromagnetic tag	RR	Loop antenna + split-ring resonator	50 × 50 mm conformal sensor, inkjet and extrusion printing on polyimide and PET ^17^ substrate, sensitivity 1.7 MHz/mm, validated vs. BIOPAC belt, 1 subject, depth correlation (0.991–0.996), RR correlation 0.993	[41]
Textile- integrated antenna	RR	Embroidered loop antenna	17 × 11 mm compact 2.4 GHz BL ^18^ transmitter, antenna resonance modulated by mechanical stretching, sensitivity 96.7%, validated vs Biopac MP36, LoAs −7.3–10.6 rpm, RMSE 4.7 rpm	[168]

^1^ Respiration rate, ^2^ tidal volume, ^3^ high intensity intermittent training, ^4^ Limit of agreement, ^5^ electromyography, ^6^ obstructive sleep apnea, ^7^ root mean square, ^8^ area under the curve, ^9^ convolutional neural network, ^10^ long short-term memory, ^11^ electrocardiography, ^12^ deep learning, ^13^ Leave-One-Subject-Out, ^14^ mean absolute percentage error, ^15^ mean absolute error, ^16^ respiration per minute, ^17^ polyethylene terephthalate, ^18^ Bluetooth.

### 2.5. Conclusions

Overall, chest motion sensors, bioimpedance systems, and IMU/SCG-based approaches represent the three dominant wearable strategies for respiratory monitoring in dynamic conditions, each offering different compromises between physiological relevance, robustness, wearability, and computational complexity. Chest belts and patch-based systems remain among the most physiologically intuitive and accurate approaches because they provide direct access to the respiratory waveform. In their simplest single-band form, they are primarily suited for RR estimation, whereas reliable V_T_ assessment usually requires dual-band or multi-point measurements capturing both thoracic and abdominal motion [92]. Although respiration belts are highly effective for waveform tracking [50], disagreement with airflow-based reference methods arises from the fact that they measure only regional surface displacement rather than the full respiratory volume. Consequently, V_T_ estimation strongly depends on sensor placement, sensing area, number of sensing elements, and subject-specific calibration [54,95]. The growing importance of textile-integrated respiratory sensing is reflected by a systematic review [169], which highlighted the rapid expansion of smart textile research, particularly in piezoresistive, capacitive, and fiber-based sensing technologies for continuous breathing monitoring. Across the reviewed studies, the sensing principle itself often appeared less critical than factors such as mechanical coupling, electrode or sensor positioning, stability of body contact, and signal-processing methodology [57]. This is particularly evident under unconstrained conditions, where motion artifacts, posture transitions, irregular breathing, speech, and upper-body movement substantially increase variability compared with controlled or paced-breathing scenarios [51,90,95,98].

Compared with purely mechanical sensing, bioimpedance provides a more direct physiological representation of respiratory-related lung volume changes and therefore appears particularly attractive for V_T_ monitoring [107]. Measurement quality, however, strongly depends on electrode configuration and on how effectively the injected electrical field traverses lung-representative thoracic regions [106,108]. Larger electrode spacing and multichannel configurations generally improve physiological sensitivity [110], while several studies suggest that more distant placements may improve long-term repeatability [115,116,119]. In practical ambulatory conditions, bioimpedance remains sensitive to motion artifacts, variability in electrode–skin contact, skin impedance changes, and EMG interference, requiring advanced filtering, adaptive modeling, and increasingly also ML-based signal-quality evaluation and artifact suppression [112,113,114]. An important practical advantage is that bioimpedance can often share electrode configurations with ECG acquisition, making it particularly suitable for multimodal wearable systems aimed at simultaneous cardiorespiratory monitoring [120,121,122,123,124,127,128].

IMU and SCG approaches generally achieve lower standalone accuracy for RR and V_T_ estimation than chest belts or bioimpedance systems, mainly because respiratory micromotions are easily masked by locomotor activity. Their major advantage, however, lies in the direct capture of posture and body movement, which makes them highly valuable as complementary modalities for motion compensation and contextual awareness in hybrid wearable systems. Recent studies show that advanced signal-processing pipelines incorporating quaternion modeling, adaptive filtering, ML, DL [149], and AI-based approaches [170,171] can substantially improve robustness, particularly when longer analysis windows are available. Owing to their low cost, small dimensions, low power consumption, and straightforward electronic integration, IMU sensors remain highly attractive for telemedicine and multimodal wearable monitoring, as discussed further in Section 4. Nevertheless, increasingly sophisticated algorithms also raise computational demands, which may complicate real-time implementation in low-power wearable devices.

## 3. Indirect (Derived) Methods

Indirect respiratory monitoring methods leverage physiological modulations embedded in routinely acquired cardiovascular signals, enabling respiration estimation without dedicated sensors. This paradigm is particularly attractive for wearable systems, where minimizing sensor complexity, power consumption, and user discomfort is critical. In this context, Charlton et al. [172] and Ponsiglione et al. [173] compared ECG-derived and PPG-derived respiratory estimation approaches and reached a consistent conclusion: ECG-derived respiration is generally more accurate and robust than PPG-based estimation. In the best dynamic scenario, the first study indicated that PPG-based estimation was roughly 30% less accurate than ECG-derived respiration, whereas the second reported an even more pronounced degradation, with PPG performance being approximately five times worse. Together, these studies highlight that although both modalities can support indirect respiratory monitoring, ECG-derived methods currently provide higher reliability, while PPG remains attractive mainly because of its superior practicality and widespread integration in wearable devices.

### 3.1. ECG-Derived Respiration

**Figure 5:**
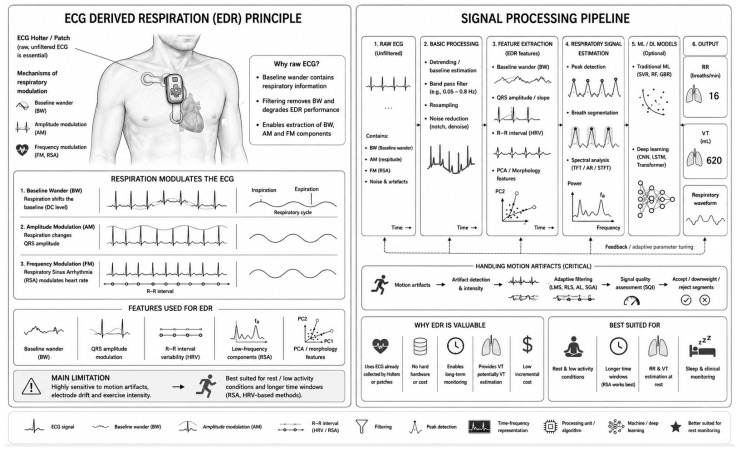
The principle of ECG-derived respiratory monitoring. The figure was developed through an iterative process combining the author’s manual drawing and AI generative tools, including ChatGPT (GPT-4o; OpenAI, San Francisco, CA, USA) and Gemini (Gemini 3 Flash Image; Google, Mountain View, CA, USA).

Respiration induces systematic modulations in the ECG. These modulations result from a combination of physiological and mechanical mechanisms, including changes in thoracic impedance associated with respiration, cyclic displacement of ECG electrodes relative to the heart, and autonomic modulation of heart rate. Together, these effects form the basis of ECG-derived (EDR) approaches to respiration (Figure 5) (Table 5) [174,175,176]. Most EDR methods, especially temporal approaches, extract respiration-related features contained in the ECG signal, such as baseline wandering, QRS complex amplitude modulation, and HRV. Frequency-domain methods isolate low-frequency components corresponding to typical respiratory frequencies and estimate the RR from dominant spectral peaks [177,178]. However, EDR performance degrades during physical activity due to motion artifacts, electrode drift, and exercise-induced changes in heart rate dynamics [16]. Several algorithms have been proposed to address these limitations, combining multiple EDR features or using advanced signal processing techniques [179,180,181,182,183].

#### 3.1.1. RR Estimation

The most common ECG-derived approach for RR estimation is based on respiratory sinus arrhythmia (RSA), which exploits respiration-related modulations in cardiac rhythm. Several studies have shown that this principle can provide reasonably accurate RR estimates even during physical activity, although performance generally declines as motion intensity increases. For example, treadmill-based validation on runners [184] showed that ECG-derived RR can be estimated with acceptable error when appropriate preprocessing and frequency-tracking methods are applied. They specifically applied bandpass filtering with short-time Fourier transform (STFT), and relative RR interval transformation with harmonic frequency tracking. Similarly, Gronwald et al. [185] evaluated EDR based on HRV and R-wave amplitude variability during treadmill running and reported good agreement with the reference under resting conditions, whereas accuracy deteriorated during exercise. These findings confirm that ECG-derived RR is feasible in motion, but remains sensitive to changes in exercise intensity, signal quality, and breathing irregularity.

To improve robustness, a wide range of EDR signal extraction strategies has been proposed. Lenis et al. [186] proposed that optimal linear combinations of EDR methods outperform individual approaches. Comparative studies have analyzed a wide range of EDR characteristics derived from QRS complex morphology [187,188], T-wave characteristics, HRV, and low-frequency spectral components, consistently identifying QRS complex-based methods, such as principal component analysis (PCA), R-peak amplitude, QRS complex integral, and RS complex amplitude, as among the most reliable. Langley et al. [189] introduced PCA as a superior approach for deriving RR from ECG amplitude variations, showing improved robustness in tracking beat-to-beat morphological changes compared with traditional RSA-based methods. This line of work was extended by Varon et al. [190], who performed a comprehensive comparative analysis of ten EDR methods using single-lead ambulatory ECG recordings. Their results showed that methods based on QRS complex slope, particularly slope range, provided the highest stability and accuracy, outperforming amplitude-based approaches in noisy and motion-corrupted conditions. In a similar effort to improve real-time applicability, Krishnapriya et al. [191] proposed a computationally efficient time-domain algorithm based on mean, prominence, and distance (MPD) parameters for respiratory peak detection in EDR signals. When evaluated on benchmark datasets and real-time recordings from subjects performing dynamic activities, the method outperformed several conventional time–domain approaches. An important practical advantage of such time–domain methods is that they process signals directly, making them attractive for low-power and resource-constrained wearable implementations [16]. More recently, EDR research has also moved toward AI-driven architectures. Qi Zhao et al. [192] proposed an improved transformer-based model for RR prediction from ECG and PPG signals, using initial feature extraction blocks followed by deep temporal modeling. Trained and evaluated using subject-level ten-fold cross-validation on the combined BIDMC and CapnoBase datasets, the model outperformed five commonly used deep-learning baselines by reducing MAE and improving correlation with the reference. Addressing the susceptibility of EDR respiration to ambulatory noise, Saha et al. [193] proposed a lightweight frequency demodulation framework integrating signal quality assessment. The method extracts respiratory-induced frequency variations and estimates RR via Fourier analysis. Validated on the CapnoBase and BIDMC datasets (achieving MAEs of 5.01 and 5.37 rpm, respectively), the integrated signal quality-aware (SQA) crucially reduced clinical false alarm rates by 84.85%. Due to its low computational complexity and simplified pipeline, the framework is highly optimized for real-time wearable monitoring.

This translational direction is also reflected in recent hardware-integrated EDR systems. The Frontier X2 chest strap (Fourth Frontier Technologies Ltd., London, UK) [194] combines continuous ECG waveform acquisition with embedded EDR-based RR monitoring, enabling real-time respiratory tracking together with cardiac workload assessment during both exercise and sleep. In parallel, Fan et al. [195] designed and fabricated a dedicated ultra-low-power processor for EDR estimation in wearable applications. Using 55 nm technology, they implemented QRS detection together with adaptive threshold-based EDR extraction, achieving estimation errors of 0.73 on the CEBS database and 1.2 on the MIT-BIH polysomnography database.

#### 3.1.2. V_T_ Estimation

All research so far has predominantly focused on RR estimation. However, some researchers have also explored the more challenging task of V_T_ estimation. Lazaro et al. [196] validated a wrist-worn device capable of deriving not only RR but also V_T_ from ECG-related features, including QRS slope range, R-wave angle, and R–S amplitude. In static conditions, the proposed approach demonstrated a strong linear relationship with reference spirometry. Yang et al. [197] investigated the feasibility of V_T_ estimation using clinical ICU data. EDR waveforms were compared with impedance-based respiration references using both linear regression and DL approaches. While short-term correlations between V_T_ and respiration waveforms were relatively strong (r = 0.78–0.96), performance substantially deteriorated over longer recordings due to noisy ECG conditions. Population-level V_T_ prediction showed limited performance (R^2^ = 0.17), whereas constrained subject-specific analyses achieved considerably higher accuracy (R^2^ = 0.84–0.94). This line of research was later extended to dynamic conditions by Milagro et al. [198], who investigated V_T_ estimation during treadmill exercise. Their approach exploited several ECG-derived features previously linked to V_T_, including EDR, HRV, and RR, which were combined in a subject-specific linear model. The model was calibrated using maximal treadmill-test data and subsequently applied to submaximal exercise.

#### 3.1.3. Location Optimization

Klum et al. [199] described optimal sensor placement using a chest ECG sensor. By implementing three EDR algorithms, derived from HRV, QRS amplitude, and linear PCA, they again found that linear PCA significantly outperformed the other methods, particularly in maintaining signal integrity across various postures. Their study also confirmed that specific electrode positions yield significantly higher signal correlations, directly supporting the findings of our previous research [200], where we optimized electrode placement for miniaturized sensors and demonstrated that, despite low inter-electrode separation, reliable EDR signals can be obtained by leveraging respiration-induced changes in thoracic impedance distribution that modulate QRS complex amplitude.

**Table 5:** ECG-derived respiratory monitoring.

Sensor Type	Application	Sensing Element/Algorithm	Key Parameters	Ref.
ECG ^1^ dataset	RR ^2^	VORTAL dataset	Validated vs. oral–nasal pressure, 39 subjects, supine and exercise, sampling rate 500 Hz, AM ^3^, FM ^4^ and BWM ^5^ method for signal extraction, SQI ^6^ + fusion technique, TD ^7^ based RR MAE ^8^ 6.4 rpm ^9^ (zero crossing method), 4.7 rpm (Count-Orig approach), bias 0 rpm	[172]
ECG datasets	HR ^10^, RR	EDR ^11^	ECG-based (R-peak, QRS area, up-slope, down-slope), 30 s-time window: iAMwell dataset (running) MAE 0.99–1.04 rpm, Capnobase dataset MAE 3.07–3.74 rpm	[173]
ECG dataset	ECG, RR	Frequency EDR	CapnoBase dataset, extract QRS + compute PBA ^12^ + filter (0.07–0.5 Hz), MAE 0.5 rpm, TD analysis, MAE 6 rpm	[174]
ECG + dataset	RR, HR	EDR, PAV ^13^ Biopac MP45	Fantasia database (*n* = 20) + real-time ECG (*n* = 10), sampling rate 1 kHz, validated vs. chest belt, EDR vs. PAV method, MAE ± 0.57 rpm (EDR), ±0.7 rpm (PAV)	[178]
ECG dataset	RR, HR, respiration waveform	EDR, EMD ^14^	MATLAB R2026a algorithms, Fantasia database, validate vs. respiratory stretch sensor, 40 subjects, MAE (0.89–1.07 rpm), percentage error 4.78–6.60%	[180]
Chest belt	RR, HR	EDR, RSA ^15^	31 subjects, running on a treadmill with a gradual increase in power until exhaustion, HR from a Polar H10A, validated vs. Cosmed Quark CPET system, 18 methods, best results: Bandpass in combination with STFT ^16^ (MAPE ^17^ 5.5%) and relative transformation of RR intervals with harmonic frequency tracking (MAPE 7.6%)	[184]
Chest belt	RR	EDR, HRV ^18^ + R-wave amplitude variability	Movesense Medical sensor, 15 subjects during treadmill running, validated vs. metabolic cart, correlation 0.80, ICC ^19^ = 0.87, mean difference: −0.5 ± 2.4 rpm (rest), 1.8 ± 4.4 rpm (exercise), LoAs ^20^: −5.2–4.2 rpm (rest), −6.9–10.4 rpm (exercise), MAE 1.6 ± 1.8 rpm (rest), 3.1 ± 3.6 rpm (exercise)	[185]
ECG datasets	RR	EDR, RSA	Fantasia (n = 40), MIT-BIH Polysomnographic dataset (n = 18), sampling rate 250 Hz, optimal linear combination of EDR methods (PCA ^21^, R peak, QRS integral, RS amplitude T peak, T integral), time window 20 s, fixed coefficient vector MCCC ^22^ 0.8 (Fantasia), 0.9 (MIT-BIH)	[186]
ECG	RR	ECG amplitudes, PCA	ECG (500 Hz), paced and normal breathing, validated vs. magnetic displacement sensor, 20 subjects, PCA-based algorithm, coherence < 0.05, correlation < 0.0001	[189]
ECG datasets	RR, respiratory waveform	EDR	3× datasets: Fantasia (n = 40), drivers (n = 16) and PSG ^23^ (n = 100), validated vs. chest belts and airflow, RS complex + QRS slope best for respiratory waveform reconstruction	[190]
ECG + dataset	RR	EDR (AFE ^24^ AD8232), TDA ^25^	MPD ^26^ algorithm optimized on Physio Net dataset, MPD vs. count origin method, MAE 3.66 rpm (MPD), 5.09 rpm (Count-Orig), MAPE 23.69% (MPD), 32.76% (Count-Orig), MPD in dynamic activities MAE 1.53 rpm, MAPE 7.25%	[191]
ECG dataset	RR	ECG + PPG ^27^, transformer-based model	Transformer-based model, ECG + PPG fusion, CapnoBase (n = 42) BIDMC (n = 53) datasets, sampling rate 125 Hz, BIIRF ^28^ extract RR, LoA 95%, MAE: 1.33 rpm (BIDMC), 0.96 rpm (Capnobase), 1.20 rpm (combined training), LoA: −3.46–3.71 rpm (BIDMC), −2.87–3.11 (Capnobase), −3.25–3.97 (combined), PCC ^29^ 0.85	[192]
ECG datasets	RR	EDR	Signal quality-aware frequency demodulation algorithm, MAE 5.01 rpm (CapnoBase), 5.37 rpm (BIDMC), signal quality assessment accuracy 85.25%	[193]
Chest-worn	ECG, HR, HRV, RR, VO_2_ max ^30^	ECG electrodes, IMU ^31^	Continual ECG, strain metrics, training load, recovery metrics, step cadence, activity tracking, Bluetooth, IP67, 14-day battery life	[194]
EDR chip, datasets	RR, HR	EDR	55 nm fabricated processor, refractory period, adaptive threshold EDR, QRS detection accuracy 99.18%, tested on 2 datasets, MAE 0.73 rpm (CEBS ^32^), 1.2 rpm (MIT-BIH)	[195]
Armband	RR, V_T_^33^	EDR	EDR: QRS slope, R-wave angle, R-S amplitude, PCA, breathing exercise, validated vs. spirometry, V_T_ and EDR amplitude correlation 0.045–0.85, MLR ^34^ model correlation 0.82–0.92	[196]
ECG	V_T_	EDR	DL ^35^ + linear regression, 90 ICU ^36^ subjects, validated vs. impedance respiratory waveform, correlation 0.78–0.96, population-level performance 0.17, subject-specific performance 0.84–0.94	[197]
Multi-lead ECG	V_T_	EDR	Treadmill exercise, 25 subjects, validated vs. spirometry, sampling rate 1000 Hz, subject-specific linear model (EDR, HRV, RR), relative fitting error < 14%, V_T_ relative error 10.23–22.72%	[198]
Chest patch system	RR, HRV	EDR	ECG at 27 differential chest positions, 3 EDR algorithms EDR, HRV, EDR amplitude, linear PCA, lowest RR mean error: 0.68 ± 0.33 rpm (F.III electrode position)	[199]

^1^ Electrocardiography, ^2^ respiration rate, ^3^ amplitude modulation, ^4^ frequency modulation, ^5^ baseline wander modulation, ^6^ signal quality index, ^7^ time domain, ^8^ mean absolute error, ^9^ respirations per minute, ^10^ heart rate, ^11^ ECG-derived respiration, ^12^ peak-to-baseline amplitude, ^13^ peak-to-peak amplitude variation, ^14^ empirical mode decomposition, ^15^ respiratory sinus arrhythmia, ^16^ short-time Fourier transform, ^17^ mean absolute percentage error, ^18^ heart rate variability, ^19^ intraclass correlation coefficient, ^20^ limits of agreement, ^21^ principal component analysis, ^22^ multiple correlation coefficient, ^23^ polysomnography, ^24^ analog front-end, ^25^ time –domain analysis, ^26^ modified peak detection, ^27^ photoplethysmography, ^28^ band-limited instantaneous respiratory frequency, ^29^ Pearson correlation coefficient, ^30^ maximal oxygen uptake, ^31^ inertial measurement unit, ^32^ combined measurement of ECG, breathing and seismocardiograms dataset, ^33^ tidal volume, ^34^ multiple linear regression, ^35^ deep learning, ^36^ intensive care unit.

### 3.2. PPG-Derived Respiration

**Figure 6:**
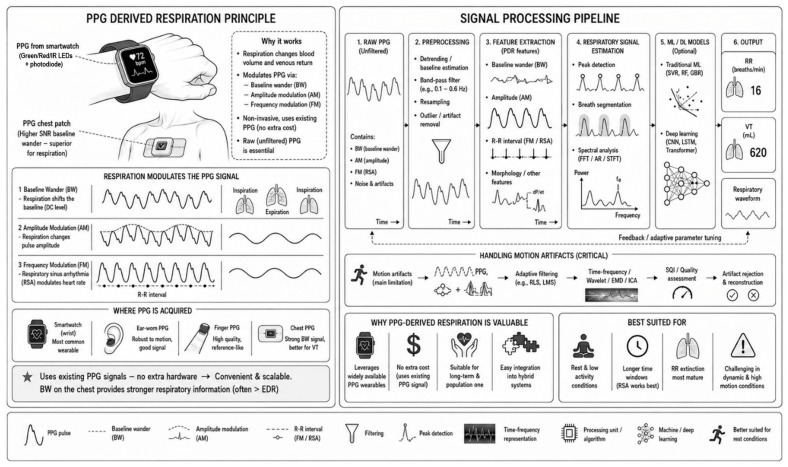
The principle of PPG-derived respiratory monitoring. The figure was developed through an iterative process combining the author’s manual drawing and generative AI tools, including ChatGPT (GPT-4o; OpenAI, San Francisco, CA, USA) and Gemini (Gemini 3 Flash Image; Google, Mountain View, CA, USA).

PPG is an optical measurement of changes in blood volume of peripheral tissues and is the most widely used physiological signal in current wearable devices like smartwatches, fitness trackers, and self-adhesive skin patches. Respiratory activity, like ECG, also systematically modulates the PPG signal through several mechanisms. This enables indirect estimation of respiratory parameters without special respiratory hardware (Figure 6) (Table 6). These effects arise, among others, from respiration-induced changes in venous return, intrathoracic pressure, and autonomic regulation, which together affect the pulse amplitude, baseline shift, and temporal characteristics of the PPG waveform [16,182,201].

#### 3.2.1. RR Estimation

Signal processing approaches commonly use envelope extraction, Hilbert transform-based analysis, adaptive filtering, or time-frequency methods to reconstruct the respiratory waveform and estimate RR. Under controlled or low-motion conditions, these techniques provide reliable results [202,203]. In commercial wearables, the WHOOP algorithm [204] is often incorporated as a standard solution. However, under dynamic conditions, PPG signals are highly susceptible to motion artifacts, mainly caused by sensor displacement, tissue deformation, and variations in optical coupling. These artifacts often dominate respiratory-related modulations and substantially reduce estimation accuracy [205]. For example, Motin et al. [206] achieved an MAE of 3.05 rpm using ensemble empirical mode decomposition.

To improve robustness, several studies have focused on combining multiple signal processing strategies or incorporating signal quality assessment. A method-fusion framework integrating several beat-detection- and waveform-morphology-based RR estimation approaches was proposed by Koumpouzi et al. [207], evaluated on the CapnoBase benchmark respiration database, and the fused PPG approach outperformed the individual methods. Similarly, Pimentel et al. [208] proposed a robust fusion-based technique incorporating probabilistic estimation for clinical RR monitoring. Cernat et al. [209] estimated RR from both infrared and green PPG channels and developed a real-time fusion model combining five PPG features. Along similar lines, Suleman et al. [210] demonstrated the feasibility of estimating respiratory events from PPG across multiple body positions, supporting low-complexity and location-independent RR monitoring. Dai et al. [211] further advanced this concept with “RespWatch,” a smartwatch-based system that combines a signal-processing RR estimator optimized for low-noise conditions with a CNN-based estimator designed for severe motion. An adaptive hybrid estimator dynamically switched between the two according to an Estimation SQI.

Several studies also demonstrated that these approaches can be translated into practical wearable devices under demanding conditions. Muller et al. [212] used the CardioWatch 287-2 (Corsano Health, Den Haag, The Netherlands) during high-intensity cycling. Using an ECG patch (Vivalink) as the reference, the PPG-based algorithm demonstrated acceptable accuracy even during intense exercise. Similarly, Eisenkraft et al. [213] clinically validated a wearable RR monitoring device based on the BB-613P sensor platform (Biobeat Technologies Ltd., Petah Tikva, Israel) [214]. Across three independent studies, the system showed high correlations with the reference and excellent Bland–Altman agreement, with biases remaining below 0.1 rpm. Zhao et al. [215] proposed “BreathAnalyzer,” a system implemented on commercial smartwatches that addresses the common limitation of weakening RSA-related signatures at high RR. Rather than relying on a single spectral component, the method integrates features from frequency, time, and nonlinear Poincaré domains. To accommodate both motion artifacts and wearable computational constraints, the system employs a lightweight tree-based learning model. Extensive evaluation showed that this multidomain approach significantly outperformed several state-of-the-art techniques, particularly during activities associated with high RR. These findings indicate that, despite the known sensitivity of PPG to motion, appropriately designed wearable systems can still achieve clinically relevant RR estimation performance in real-world scenarios.

More recently, machine learning approaches have gained traction as a means of improving robustness in dynamic and motion-corrupted scenarios [216]. Stankoski et al. [217], using the XGB algorithm, achieved an MAE of 1.38 rpm and a Pearson’s correlation coefficient of 0.86. Chin et al. [218], employing the RR estimation toolbox together with convolutional and LSTM layers, reported an MAE of 2 rpm. Baker et al. [219] combined signal quality quantification with several neural networks and achieved an MAE of 0.638 rpm. Shuzan et al. [220] presented an ML-based RR estimation framework incorporating motion artifact correction and PPG features, where feature selection was used to reduce computational complexity and overfitting. Their best-performing Gaussian process regression model achieved an RMSE of 2.63 rpm, an MAE of 1.97 rpm, and a two-standard deviation of 5.25 rpm. Ganeshmurthy et al. [221] proposed an RR estimation method based on optimization of temporal segmentation windows and preprocessing, achieving strong performance on both the BIDMC and TMCH datasets. Lee et al. [222] further improved estimation by combining autocorrelation-based spectral features with nonparametric bootstrap feature generation and Gaussian process regression, while also providing uncertainty estimates.

Additional conventional algorithmic studies include Karlen et al. [223], who estimated RR using the Incremental-Merge Segmentation algorithm and fast Fourier transform (FFT), Schäfer and Kratky [177], who compared multiple approaches and reported the best-performing method, and Dubey et al. [224], who applied a spectral kurtosis-based method for RR estimation.

The most advanced recent studies have moved toward end-to-end deep learning and signal reconstruction. Ravichandran et al. [225] proposed a ResNet-based model that directly reconstructs the respiratory waveform from PPG signals, achieving high cross-correlation and low reconstruction error across two benchmark datasets. A similar ResNet-based strategy for RR estimation was later adopted by Bian et al. [226], who showed that training with augmented synthetic data improved performance by approximately 34%. Aqajari et al. [227] applied CycleGAN to reconstruct clean respiratory signals from PPG, while Zargari et al. [228] further demonstrated CycleGAN-based correction of motion-corrupted PPG without relying on accelerometer input, achieving substantial improvement in artifact suppression (9.5–times).

Recent advancements in PPG-based respiratory monitoring leverage sophisticated AI to overcome inherent sensor limitations, such as signal degradation, computational demands, and dataset imbalances. To address signal quality degradation under free-living conditions, Pham et al. [229] introduced “CP-PPG,” an adversarial generative model designed to correct waveforms distorted by variable skin–sensor contact pressure. By stabilizing morphology-related amplitude distortions, this framework improved RR estimation accuracy by approximately 6.85%. Tackling motion artifacts, Rajendran et al. [230] proposed a heuristic-aided ensemble learning framework that combines multilayer perceptron (MLP), AdaBoost, and attention-based LSTM (A-LSTM) architectures. Optimized by the Advanced Golden Tortoise Beetle Optimizer (AGTBO), this multimodal approach achieved up to 96% accuracy in simultaneous RR and SpO_2_ estimation, outperforming conventional MLP (90%), AdaBoost (92%), A-LSTM (92%), and hybrid MLP-ADA-A-LSTM (94%) approaches. Transitioning from manual feature engineering to end-to-end DL, Shuzan et al. [231] developed “PPG2RespNet,” a U-Net-inspired architecture with hierarchical skip connections for direct respiratory waveform reconstruction. Validated across multiple datasets (BIDMC, VORTAL, CapnoBase), it achieved high Pearson correlation coefficients (0.94–0.96) and extremely low MAEs of 0.11–0.69 rpm.

Miao et al. [232] introduced “RespDiff,” an end-to-end multi-scale recurrent neural network (RNN) diffusion model. Notably, this framework bypasses the need for handcrafted feature extraction or the exclusion of low-quality signal segments, significantly enhancing its viability for real-world wearable applications. By integrating multi-scale encoders with a bidirectional RNN and a specific spectral loss term, the model effectively captures temporal respiratory dynamics while maintaining high waveform fidelity. Validation on the BIDMC dataset demonstrated superior respiratory rate estimation with an MAE of 1.18 rpm, outperforming conventional methods with results between 1.66 and 2.15 rpm. For power-constrained wearables, Yang et al. [233] presented a highly energy-efficient alternative using spiking neural networks (SNNs). By directly converting PPG segments into sequential spike events, the model preserved temporal dynamics while substantially reducing computational overhead, maintaining competitive RR estimation accuracy (MAEs of 1.15–1.37 rpm). Finally, addressing the critical issue of imbalanced training data, Lee et al. [222] proposed an imbalanced power spectral generation (IPSG) framework combined with Gaussian Process Regression (GPR). By generating artificial spectral feature curves, the model improved learning for underrepresented abnormal respiratory conditions. Validated on the BIDMC dataset, it achieved MAEs between 0.79 and 1.47 rpm, while distinctively providing uncertainty estimates to quantify prediction reliability for clinical interpretation.

#### 3.2.2. V_T_ Estimation

Unlike RR estimation, PPG-based estimation of V_T_ has been investigated only in a limited number of studies. Early physiological studies demonstrated that respiratory modulations in the PPG waveform are influenced by V_T_ and respiration pattern, indicating the theoretical feasibility of V_T_ tracking from PPG. More recent wearable work, such as wearable PPG systems, has extended this concept toward continuous monitoring of breathing phase and tidal volume, although this area remains much less mature than PPG-derived RR estimation [234]. More recently, Romero et al. [235] proposed OptiBreathe, a wearable PPG-based system for estimating multiple respiratory biomarkers, including RR, breathing phases, and V_T_. Their pipeline takes into consideration three modulations: respiratory-induced intensity variation, AM, and FM. In validation against spirometry, during static testing, the device achieved a best MAE of 1.96 rpm for RR and a best subject-averaged MAPE of 17% for V_T_, suggesting that PPG may also support volumetric respiratory monitoring. Unfortunately, we did not find tests in a dynamic environment.

**Table 6:** PPG-derived respiratory monitoring.

Sensor Type	Application	Sensing Element/Algorithm	Key Parameters	Ref.
PPG ^1^ dataset	RR ^2^	VORTAL dataset	Validated vs. oral–nasal pressure, 39 subjects, supine and exercise, sampling rate 500 Hz, AM ^3^, FM ^4^ and BWM ^5^ method for signal extraction, SQI ^6^ + fusion technique, TD ^7^ based Count-Orig approach LoAs ^8^ −5.1–7.2 rpm ^9^, bias 1 rpm	[172]
PPG datasets	HR ^10^, RR	CapnoBase, iAMwell datasets	PPG-based on FM, AM, 30 s-time window: MAE ^11^ 5.10–5.12 rpm (iAMwell dataset—running), 10.7–13.9 rpm (Capnobase dataset)	[173]
PPG dataset	RR	VORTAL dataset, intrinsic modes	39 subjects during rest (supine), Recursive Bayesian Tracking, intrinsic modes, time-frequency spectra, extraction of amplitude variability, VORTAL database, WSST ^12^ MAE 2.33 rpm, ME ^13^ 1.15 rpm	[202]
Laboratory setup	RR, HR	PPG Nnormalized LMS ^14^	In vitro PPG, skin perfusion phantom, motion artifacts correlation, measuring via self-mixing interferometry, artifact reduction −9.9 dB	[205]
PPG sensors	RR	PPG EEMD ^15^	PPG BIOPAC MP150, 10 subjects, 5 activities, validated vs. chest belt, MAE: 3.16 rpm (sitting), 3.02 rpm (standing), 3.01 rpm (walking), 3.07 rpm (stairs climbing), 3.18 (running)	[206]
PPG dataset	RR	CapnoBase dataset	42 subjects, PPG beat segmentation, peak extraction, 60 s-time window, RMSE ^16^ 3.4 rpm	[207]
PPG datasets	RR	CapnoBase, MMIC II datasets segmentation	PPG modulations exhibited, CapnoBase (300 Hz), MMIC II conventional BioZ ^17^ (125 Hz), AM + FM + BW ^18^ extraction, segmentation algorithm, Gaussian process, MAE 2.7 rpm (2.1–3.2 rpm)	[208]
PPG sensor	RR	Real-time PPG	IR ^19^ PPG signal, 12 features, determine PAV ^20^, PWV ^21^, shimmer sensor node, sample rate 102.4 Hz, simpler FFT ^22^, Error < 2 rpm (in range 6–30 rpm), RMSE: 0.77–1.41 rpm (low RR), 5.86–17.34 rpm (high RR)	[209]
PPG datasets	RR	CapnoBase, BIDMC datasets	FFT analysis and peak detection, MAE 2.14 ± 5.59 rpm (CapnoBase), 1.59 ± 3.21 rpm (BIDMC)	[210]
RespWatch	RR, HRV	PPG watch CNN ^23^ model	Gen 4 Explorist watch, sampling rate 50 Hz, validated vs. Vernier belt, 32 subjects, RR during high activity, new estimation quality index, MAE 0.9–2 rpm (based on motion intensity)	[211]
CardioWatch	RR, HR	PPG	HIIT RR, CardioWatch, 35 subjects, validated vs. Vivalink ECG, during high activity, average RMSE 2.13 rpm (Rest: 1.5 rpm, moderate motion: 2.4 rpm), bias 0.09 rpm, LoAs 4.28–4.09 rpm	[212]
Wrist and chest monitor	RR, HR	PPG Biobeat BB-613WP	3 studies: 35 subjects, 18 ventilated, 92 COVID-19 patients, validated vs. ventilatory system, PPG-enhanced by skin tone and BMI ^24^, Pearson’s correlations ≤ 0.05, correlation 0.991, 0.884, 0.888, resp. *p* < 0.001, 95% LoA ± 2.3 rpm	[213]
Watch	RR	PPG	RSA ^25^, single spectrum or raw signal, 3× based learning model, RespBoost (BreathAnalyzer) model, high RRs improvement 35.37–80.42%, MAE during sport: 3.94 rpm (BreathAnalyzer), 13.3 rpm (HeartPy), 6 rpm (Respwatch), 8 rpm (WearBreathing)	[215]
PPG sensors	HR, RR	PPG	Machine-aided Signal quality assessment applied to PPG, 116 subjects, MAE for HR 3.06 bpm ^26^, for RR 1.36 rpm, Predicting hypertension +24%	[216]
Head worn PPG sensor	RR	PPG, accelerometer, XGB ^27^	VR headset, EmteqPRO biometric mask, controlled breathing, 37 subjects, XGB algorithm, MAE 1.38 rpm	[217]
PPG finger probe dataset	RR	PPG, CNN-LSTM ^28^ model	BIDMC datasets–ECG, PPG, BioZ, CapnoBase dataset, 42 subjects, resampled 125 Hz to 30 Hz, CNN-LSTM model MAE 2.02 rpm, CapnoBase MAE 1.24 rpm	[218]
PPG finger probe datasets	RR	PPG, ECG BiLSTM ^29^ network	MIMIC-III database, BW, AM, FM, 3 RR segment lengths, signal quality index, respiratory quality index reduced MAE 36.89%	[219]
Wrist monitor	RR, HRV	ECG, PPG	VORTAL dataset, different ML ^30^ (SVR ^31^, GPR ^32^), sampling rate 500 Hz, 758 PPG segments, MAE 1.91 rpm, RMSE (2SD 2.66 and 5.30 rpm)	[220]
PPG datasets	RR	TMCH + BIDMC datasets	Preprocessing: Chebyshev filtering, signal quality index, 2× datasets: TMCH (n = 524), MAE 0.73 rpm, RMSE 0.93 rpm in 40s window, BIDMC (n = 53), MAE 2.07 rpm, RMSE 1.95 rpm in 120 s window	[221]
PPG sensor	RR, HR	Real-time PPG IMS ^33^ algorithm	Real-time frequency, intensity and amplitude extracted via IMS, respiratory-induced frequency variation obtained using FFT, 42 subjects, algorithm in a mobile phone, RMSE 3 ± 4.7 rpm	[223]
Wrist band	RR	PPG	Smartphone processor, 556 nm LED ^34^, ELM ^35^ regression, spectral kurtosis-based method, CapnoBase, RMSE 1.2 ± 0.3 rpm, BLE ^36^	[224]
PPG sensor datasets	RR	PPG	ResNet, Capnobase, and Vortal datasets, mean square error 0.262, 0.145 rpm, cross-correlation coefficient 0.933, 0.931 rpm	[225]
PPG sensor datasets	RR	PPG CNN ResNet	CapnoBase, BIDMC, AM, FM, BW, CNN ResNet, sampling rate 30 Hz, real data MAE 3.8 ± 0.5 rpm, synthetic data MAE 4.2 ± 0.5 rpm	[226]
PPG sensor dataset	RR	PPG	BIDMC PPG and respiration dataset (MIMIC II), sampling rate 125 Hz, CycleGAN ^37^ for signal reconstruction, 5-fold cross validation, MAE 1.9 ± 0.3 rpm	[227]
PPG + ECG dataset	HR, RR	PPG	Non-accelerometer motion artifacts removal from PPG, CycleGAN, BIDMC, MIMIC II dataset, 9.5× improved motion artifacts removal, improvement of RMSE 41×, PPE ^38^ 58×,	[228]
Wrist PPG	HR, HRV, RR, BP ^39^	PPG	Compensation of skin–sensor contact artifacts, adversarial deep generative model, CP-PPG ^40^ framework, window length: 16 s, 5-fold subject-independent cross-validation, RR improvement +6.85%, signal fidelity improvement 40% (MAE = 0.09 rpm)	[229]
PPG dataset	RR, SpO_2_^41^	PPG	Motion artifacts compensation, accuracy: 90% (MLP ^42^), 92% (A-LSTM ^43^, AdaBoost ^44^), 94% (MLP-AdaBoost-A-LSTM), 96% (AGTBO ^45^),	[230]
PPG datasets	RR, Respiratory waveform	PPG	BIDMC, VORTAL, CapnoBase, and PPG2RespNet datasets, (U-Net-inspired DL ^46^ model) algorithm, PCC ^47^ 0.94 (BIDMC), 0.95 (VORTAL), 0.96 (CapnoBase), MAE: 0.69/0.58/0.11 rpm	[231]
PPG dataset	RR, Respiratory waveform	PPG	BIDMC dataset, “RespDiff” algorithm, Diffusion model + bidirectional RNN ^48^ AI type, multi-scale encoder + BiRNN ^49^ architecture, MAE 1.18 rpm	[232]
PPG dataset	RR	PPG	BIDMC dataset, SNN ^50^ AI, input windows: 16/32/64 s, MAE: 16 s: 1.37 ± 0.04 rpm, 32 s: 1.23 ± 0.03 rpm, 64 s: 1.15 ± 0.07 rpm, energy-efficient	[233]
PPG datasets	RR	PPG	Dataset: BIDMC—53 subjects, 480 s record length, RRSYNTH–192 subjects, 210 s record length, Kaiser window algorithm with cutoff frequency 35 Hz, resampled to 5 Hz, IPSG ^51^ + GPR, uncertainty-aware ML/bootstrap augmentation AI, MAE: 0.79–1.47 rpm	[222]
PPG in ear	RR, V_T_^52^	PPG	OptiBreathe, sampling rate 100 Hz, 11 subjects, validated vs. spirometry, static test, pipeline (respiratory induced intensity variation, AM, FM), 50–100 s time window, RR MAE 1.96 rpm, averaged V_T_ MAPE ^53^ 17%	[235]

^1^ Photoplethysmography, ^2^ respiration rate, ^3^ amplitude modulation, ^4^ frequency modulation, ^5^ bandwidth modulation, ^6^ signal quality index, ^7^ time domain, ^8^ limits of agreement, ^9^ respiration per minute, ^10^ heart rate, ^11^ mean absolute error, ^12^ wavelet synchro-squeezed transform, ^13^ mean error, ^14^ normalized least mean squares, ^15^ ensemble empirical mode decomposition, ^16^ root mean square error, ^17^ bioimpedance, ^18^ bandwidth modulation, ^19^ infrared, ^20^ pulse amplitude variability, ^21^ pulse wave velocity, ^22^ fast Fourier transform, ^23^ convolutional neural network, ^24^ body mass index, ^25^ respiratory sinus arrhythmia, ^26^ beat per minute, ^27^ extreme gradient boosting, ^28^ convolutional neural network–long short-term memory, ^29^ bidirectional long short-term memory, ^30^ machine learning, ^31^ support vector regression, ^32^ Gaussian process regression, ^33^ iterative multi-scale spectrum, ^34^ light-emitting diode, ^35^ extreme learning machine, ^36^ Bluetooth low energy, ^37^ cycle-consistent generative adversarial network, ^38^ pulse rate estimation, ^39^ blood volume pressure, ^40^ contact pressure-distorted PPG, ^41^ peripheral oxygen saturation, ^42^ multilayer perceptron, ^43^ attention-based long short-term memory, ^44^ adaptive boosting, ^45^ advanced golden tortoise beetle optimizer, ^46^ deep learning, ^47^ Pearson correlation coefficients, ^48^ recurrent neural network, ^49^ bidirectional recurrent neural network, ^50^ spiking neural networks, ^51^ imbalanced power spectral generation, ^52^ tidal volume, ^53^ mean absolute percentage error.

### 3.3. Conclusions

ECG- and PPG-derived respiratory methods are used predominantly for RR estimation rather than for full reconstruction of the respiratory waveform. Their physiological basis lies in respiration-related modulation of the cardiovascular signal, most commonly through amplitude modulation (AM), frequency modulation (FM), and baseline wander (BW), which are linked to respiratory sinus arrhythmia, thoracic pressure changes, and respiration-dependent alterations in venous return and stroke volume. Because these signals reflect respiration only indirectly, they are generally more suitable for averaged RR estimation over longer analysis windows than for reliable breath-by-breath waveform reconstruction or robust V_T_ estimation.

A wide spectrum of algorithms has been proposed, ranging from simple rule-based and spectral methods to adaptive filtering, machine learning, and deep-learning frameworks. In general, algorithmic complexity tends to improve robustness, particularly under noisy or motion-corrupted conditions, but at the cost of greater computational and energy demands. Simpler approaches often rely on only one or two modulated components, frequently FM alone, which is closely related to HRV, whereas more advanced methods combine multiple respiratory surrogates to improve stability and reduce susceptibility to signal-specific artifacts. In practice, performance therefore depends strongly on the trade-off between accuracy, latency, computational burden, and suitability for continuous wearable deployment.

From the reviewed studies, ECG-derived respiration generally achieves somewhat higher accuracy and physiological consistency than PPG-derived approaches, particularly in controlled conditions [172,173]. However, both modalities possess distinct performance ceilings in dynamic scenarios. ECG is fundamentally limited at extremely high HR, where fewer cardiac cycles per breath reduce the sampling resolution needed for respiratory extraction. Furthermore, during intense physical activity, the shifting of the heart within the chest cavity varies the distance to the electrodes, which can introduce severe artifacts that completely drown out the ECG signal. Conversely, the functional ceiling of PPG is dictated by optical limitations, making it highly susceptible to strong ambient light, poor peripheral perfusion, and signal attenuation from darker skin tones. Interestingly, while the literature emphasizes PPG’s vulnerability to extremity motion artifacts, our preliminary research indicates that chest-mounted PPG can marginally outperform ECG by directly capturing mechanical thoracic expansion while avoiding wrist-induced noise. Nevertheless, because PPG offers substantially better comfort and integration potential, already embedded in most contemporary smartwatches, it is becoming the most widely adopted indirect approach in consumer wearable monitoring, despite its inherently weaker relation to respiratory mechanics.

Overall, derived methods should be viewed primarily as practical and low-hardware-cost solutions for RR tracking, while their main limitations remain reduced reliability during intense motion, dependence on cardiovascular–respiratory coupling, and limited capability for accurate V_T_ estimation [196,198,235].

## 4. Hybrid and Multisensor Approaches

**Figure 7:**
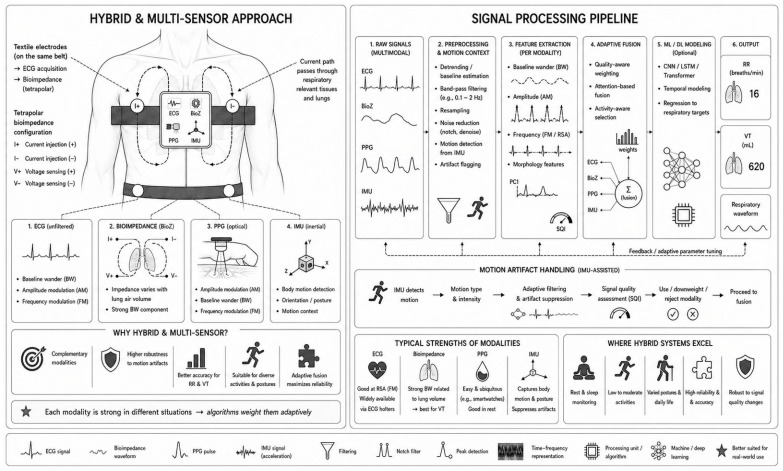
The principle of hybrid bad multisensor respiratory monitoring. The figure was developed through an iterative process combining the author’s manual drawing and generative AI tools, including ChatGPT (GPT-4o; OpenAI, San Francisco, CA, USA) and Gemini (Gemini 3 Flash Image; Google, Mountain View, CA, USA).

Hybrid and multisensor approaches (Figure 7) (Table 7) have emerged in response to the limited robustness of single-modal systems. By combining complementary sensing principles, these systems attempt to compensate for modality-specific weaknesses and improve reliability [236]. A common strategy is the integration of physiological respiratory sensing with inertial measurements. In such configurations, accelerometers or gyroscopes provide contextual information about body movement and orientation, which can be used for artifact detection, adaptive filtering, or activity-aware weighting of respiratory features [17,237].

### 4.1. Chest Belts Enhanced with IMU

One of the simplest and most practical hybrid concepts combines a chest-mounted respiratory sensor with an IMU. In these systems, the chest belt provides the primary respiratory waveform, while inertial sensing helps to identify posture changes and suppress motion-induced distortions.

An early example was presented by Wu et al. [238], who implemented a digital RIP sensor within a wireless body sensor network. Their system incorporated a textile sensor positioned on the thorax or abdomen, together with a 3-axis accelerometer used to contextualize respiratory measurements with body posture. This fusion improved the robustness of RR estimation during ambulatory use. A similar concept was further developed by De Fazio et al. [239], who designed a low-power chest band integrating a custom piezoresistive textile sensor (EeonTex LTT-SLPA-20K) with an MPU-6050 IMU. The IMU was used to mitigate motion artifacts, while onboard processing enabled local filtering and respiratory parameter extraction. The system showed strong agreement with manual breath counting in seated conditions, although performance deteriorated during walking, highlighting the persistent challenge of motion contamination.

Rather than relying solely on chest motion sensing, some systems combine inertial data with more physiologically direct modalities. Fedotov et al. [240], for example, developed a hybrid system based on bioelectrical impedance plethysmography and a 3D accelerometer. Their artifact suppression strategy combined hardware bandpass filtering with adaptive software denoising, based on an RLS algorithm derived from the Wiener–Hopf approach. Especially during higher-intensity activity, this hybrid processing substantially improved SNR and enabled reliable real-time RR estimation where conventional filtering alone was insufficient.

Several studies also extended this architecture beyond simple RR detection toward fuller respiratory characterization. Whitlock et al. [241] introduced A-Spiro, a system combining a respiratory sensor, an IMU, and lung hysteresis modeling to estimate not only RR, but also respiratory flow, V_T_, and minute ventilation. Validation across six activities demonstrated 93% accuracy for flow estimation, 94.4% accuracy for minute ventilation, and a mean RR accuracy of 96%. The reported performance suggests that hybrid motion-compensated chest systems may be particularly valuable when more detailed respiratory outputs are required. A related multisensor strategy was proposed by Zabihi et al. [242], who developed a wearable patch that fuses data from an IMU (MPU6050, TDK InvenSense, San Jose, CA, USA) with a flexible resistive pressure sensor. Their signal fusion framework incorporated FFT, short-time Fourier transform (STFT), and inertial-signal-based filtering to remove non-respiratory motion components before respiratory feature extraction. Validation against spirometry during multiple breathing maneuvers demonstrated improved robustness compared with single-sensor sensing alone.

### 4.2. EDR Enhanced with IMU

The next group comprises systems that combine EDR methods, most commonly with IMUs. In these architectures, IMU signals are again primarily used to improve robustness by identifying body motion, supporting artifact suppression, or enabling activity-aware estimation.

A representative example was presented by Alam et al. [17], who proposed a modular and generalizable framework for estimating respiratory parameters from an ECG Holter Shimmer3 ECG (Shimmer, Dublin, Ireland) and wrist-worn motion signals collected by Shimmer3 IMU (Shimmer, Dublin, Ireland). Their pipeline combined activity classification with subsequent regression models, including generalized linear models, random forest, SVM, Gaussian process regression, and neighborhood component analysis. This multimodal strategy enabled accurate estimation of both RR and V_T_. A slightly different perspective was offered by Leube et al. [243]. Interestingly, their results showed that wrist acceleration-derived respiratory proxies achieved higher phase synchronization with the reference flow signal than ECG-derived proxies and enabled more precise RR estimation. However, this advantage was largely limited to periods of minimal physical activity, such as sleep or low-movement conditions, indicating that wrist-based inertial respiration remains highly context dependent.

Several studies have focused more specifically on signal-level fusion for artifact suppression. Alhaskir et al. [244] combined ECG-derived RSA features with accelerometer signals using adaptive line enhancement, least mean squares (LMS) filtering, and singular spectrum analysis. Their work highlights how inertial information can be used not only for motion detection, but also as a direct aid in separating respiratory-related oscillations from movement-induced interference. Inertial sensing can also be incorporated as a fully complementary respiratory modality, particularly through SCG. In this context, SDR captures respiration-related mechanical modulations of chest vibrations and can therefore complement conventional EDR. A deep-learning-based example was presented by Chan et al. [245], who developed a cascaded framework for RR estimation from ECG and SCG signals acquired using a chest-worn patch. EDR and SDR signals were computed, transformed into the spectrotemporal domain, and denoised using a 2D U-Net architecture prior to feature fusion. Experimental evaluation demonstrated that multimodal fusion outperformed single-signal approaches, achieving an MAE of 0.82 rpm. Soliman et al. [246] fused EDR and SDR signals and trained an ML model for V_T_ estimation. They achieved an RMSE of 181.45 mL and a Pearson correlation coefficient of 0.61. Their results suggest that combining electrical and mechanical respiration surrogates may offer a promising route toward richer respiratory monitoring, particularly when direct airflow or volume sensing is impractical.

### 4.3. PPG Enhanced with IMU

Multimodal fusion has also been extensively explored in wrist-based and smartwatch platforms, where motion artifacts are most challenging.

A representative signal-processing approach was presented by Jarchi et al. [247], who used accelerometer signals as inputs to a normalized LMS adaptive filter to suppress motion-corrupted spectral components in the PPG waveform. The corrected signal was then reconstructed in the Hilbert domain, and RR was estimated using autoregressive spectral analysis. A related artifact-reduction strategy was proposed by Nabavi and Bhadra [248], who filtered motion-induced distortions in the PPG-derived respiratory signal using information extracted from the accelerometer spectrum combined with a band-stop filtering approach. Together, these studies illustrate the importance of IMU-assisted preprocessing as a first line of defense against motion corruption in wearable PPG systems.

More recently, the field has shifted toward data-driven smartwatch solutions. Kazemi et al. [249] used a DL framework for RR estimation from raw smartwatch PPG and accelerometer signals, combining dilated residual inception modules, multi-scale convolutions, and transfer learning from a pretrained foundation network. In a follow-up study, the same group further enhanced the framework by incorporating gyroscope data [237], demonstrating that multimodal inertial fusion can further improve RR estimation in free-living wearable scenarios.

A similar philosophy was adopted by Liaqat et al. [250], who developed WearBreathing, a smartwatch-based framework that prioritizes signal reliability over estimate density. Their method uses IMU data to reject motion-corrupted segments and applies CNN-based RR estimation only to sufficiently clean signal windows. This strategy yielded substantially lower errors than prior approaches and highlighted an important practical trade-off between estimation accuracy and temporal resolution.

Semiz [251] developed a compact multimodal patch designed for operation without conventional gel electrodes. The device integrated dual-wavelength PPG, SCG, and skin temperature sensing. The system demonstrated high accuracy for HR, HRV (MAE < 1%), and RR estimation (MAE = 1.6%). Importantly, the use of Teager–Kaiser energy operator-based SCG processing improved robustness of the respiratory extraction pipeline, supporting the feasibility of compact low-burden cardiorespiratory monitoring.

Exploring alternative anatomical sensor placements, Abdulsadig et al. [252] developed a neck-worn device integrating PPG and accelerometry. To mitigate the severe motion and postural artifacts inherent to the neck region, the framework utilizes recursive FFT-based dominance scoring combined with exponentially weighted moving average (EWMA) aggregation. Furthermore, the authors introduced rate-band estimation rather than relying solely on point estimates, thereby improving clinical interpretability. Validated under guided breathing protocols and varying oxygen conditions using an altitude generator, the system achieved RR accuracies of 88.4 ± 7.63% against reference instrumentation and 94.94 ± 3.56% relative to a visual metronome, alongside highly accurate HR extraction.

### 4.4. ECG and PPG Fusion

Fusion of ECG and PPG has also proven effective for robust RR estimation, as both modalities carry complementary respiratory information and can partially compensate for each other’s weaknesses under variable signal quality conditions. In general, such systems aim to improve estimation stability by combining multiple respiratory surrogates extracted from both waveforms.

Lin et al. [253] proposed a real-time temporal fusion framework. Their method derived six respiratory components, selected the most reliable ones using a respiratory quality index, and fused them into a single respiratory signal via component analysis. Validation on the CapnoBase and BIDMC datasets yielded MAEs of 1.39 and 3.29 rpm, respectively, corresponding to an average MAE reduction of 11.61% compared with state-of-the-art methods. A similar fusion concept was explored by John et al. [254], who developed a real-time RR estimation framework based on the discrete wavelet transform (DWT). In contrast to static fusion strategies, their method used instantaneous signal quality indices as adaptive fusion weights, allowing the system to dynamically prioritize the cleaner modality. Evaluation on the CapnoBase TBME RR dataset demonstrated excellent robustness, achieving an MAE of 0.34 rpm across a wide SNR range from −50 to 50 dB. Leet and Lee [255] introduced an uncertainty-aware framework for simultaneous RR and confidence interval (CI) estimation. The architecture integrates exact Gaussian process regression (EGPR) with multiple multilevel feature extraction (MMFE) and adaptive neighbor component analysis (ANCA) for optimized feature selection and fusion. Moving beyond conventional point predictions, the model explicitly quantifies prediction uncertainty to improve robustness under limited data conditions. Validation demonstrated high accuracy and reliable uncertainty metrics, with the PMF-EGPR configuration achieving an MAE of 0.98 rpm (CI: 4.85 rpm) and the EMF-EGPR setup yielding an MAE of 1.155 rpm (CI: 7.47 rpm).

A stronger emphasis on adaptive multimodal fusion under motion was introduced by Chan et al. [256], who addressed RR estimation during walking and exercise recovery using a chest-worn patch combining ECG, PPG, and SCG. Respiratory surrogate signals were first extracted independently from each modality, after which an adaptive channel selection strategy based on a spectral respiratory SQI identified the most reliable signal source in real time. A modality-attentive fusion framework was then used to combine spectral–temporal information, followed by a U-Net-based denoising model. This architecture achieved robust performance even under dynamic conditions and further improved after excluding low-quality segments. The fusion framework achieved an MAE of 2.21 rpm during walking, which was further reduced to 1.59 rpm after excluding low-quality segments.

A related DL perspective was proposed by Rathore et al. [257], who introduced “MRNet”, a multitasking framework for simultaneous RR estimation and respiratory waveform reconstruction from fused ECG, PPG, and IMU data. The model maintained reliable performance during walking (MAE = 2.93 rpm) and stair climbing (MAE = 3.32 rpm), and the authors explicitly emphasized the importance of architectural optimization for real-time deployment on wearable systems. Similarly, Kumar et al. [258] evaluated multiple DL architectures for multimodal RR prediction from ECG, PPG, and EMG, showing that attention-enhanced bidirectional LSTM models can provide very high accuracy (MAE = 0.24 ± 0.03 rpm), especially when longer temporal windows are available. Their findings also clearly demonstrated the trade-off between estimation accuracy and window length, which remains a key design consideration in practical wearable deployment.

An important direction within this field is represented by upper-arm multimodal wearables, which aim to balance signal quality, comfort, and unobtrusive long-term use. Branan et al. [43] developed a device integrating multi-wavelength PPG, single-sided ECG, bioimpedance, and IMU sensing. Their results showed that multimodal fusion improved the robustness and accuracy of RR and HR estimation, helping to overcome the classical accuracy–robustness trade-off associated with single-modality systems. Interestingly, their feature importance analysis revealed that bioimpedance-derived baseline wander was the dominant contributor to RR estimation, while ECG-derived features provided a smaller but complementary contribution. This is particularly notable because bioimpedance, despite not being the strongest standalone modality for all parameters, provided highly valuable respiration-specific information within the fused framework. This concept was further examined by Kurian [259] in a closely related upper-arm multimodal system using a similar sensing architecture but without IMU integration. Under controlled breathing conditions, the study systematically compared unimodal and multimodal configurations, consistently confirming the benefit of sensor fusion for RR estimation even in well-defined and relatively stable scenarios.

Pushing the boundaries of multimodal integration, recent frameworks emphasize both hardware and algorithmic fusion for robust ambulatory monitoring. Exemplifying advanced hardware integration, the chest-worn reSPIRE system [260] combines SCG, PPG, impedance pneumography, MMG, EMG, and ECG. Validated across static and dynamic conditions, including stationary cycling, the platform achieved highly accurate V_T_ estimation (R^2^ = 0.91) and respiratory muscle force tracking (Spearman ρ = 0.87). Complementing hardware advancements with algorithmic fusion, Feli et al. [261] proposed a deep MTL framework utilizing smartwatch-derived PPG, ECG, and IMU signals. By simultaneously optimizing signal quality assessment, HR, and RR estimation, the MTL architecture exploited cardiovascular-respiratory interdependencies to outperform single-task models in free-living conditions, achieving an RR MAE of 1.98 rpm.

### 4.5. Acoustic Signal Incorporation

Some hybrid systems also incorporate acoustic signals, which provide complementary information related not only to respiration rate, but also to breathing events, airflow characteristics, and cardiopulmonary abnormalities.

For example, Moon and Lee [262] developed a compact skin-adhesive device integrating acoustic lung sounds and ECG for real-time respiratory event detection, demonstrating statistically significant detection of shallow breathing and coughing events. A different application was presented by Lee et al. [263], who combined IMU data with respiratory audio acquired from smart earbuds for exercise repetition counting, achieving higher accuracy than IMU-only models across 30 exercise types. More comprehensive multimodal integration was demonstrated by Qiu et al. [264], who presented a lightweight multimodal smart chest patch integrating flexible ECG, heart sound, and respiratory sensors with a multi-criterion multimodal fusion ML model. Evaluation of 5561 recordings from 475 subjects achieved 87% accuracy for cardiopulmonary anomaly detection and demonstrated stable signal acquisition during exercise.

**Table 7:** Hybrid respiration sensors.

Sensor Type	Application	Sensing Element	Key Parameters	Ref.
Textile belt integrated in garment	RR ^1^	Digital RIP ^2^ textile sensor + 3D accelerometer	Wireless body sensor network, sensor fusion with motion data improved robustness, microprocessor MSP430F14, wireless data transmission, 800 mAh battery, 6 h battery life, 10 subjects, dynamic experiments, reliable RR	[238]
Chest belt	RR, flow rate	Piezoresistive textile sensor + IMU ^3^	IMU (MPU-6050), BLE ^4^, microcontroller SAMD21G18A for filtering, motion artifacts reduced with IMU, 6 subjects, walking: Pearson correlation coefficient 0.923, LoAs ^5^ −3.37 to +3.7 rpm (with IMU), LoAs −3.72–4.32 rpm (without IMU), onboard preprocessing and parameter extraction	[239]
Chest belt	RR	Impedance electrodes + accelerometer	Hybrid artifact suppression (active bandpass filter + software adaptive RLS ^6^ algorithm via Wiener–Hopf), sampling rate 500 Hz, 15 subjects, evaluated during rest and dynamic conditions, relative error ~1.5% (rest), ~9.2% (dynamic), increased SNR ^7^	[240]
Chest belt	RR, V_T_^8^	Capacitance sensors + IMU (A-Spiro)	Lung hysteresis modeling, evaluated across 6 activities, 20 subjects, motion correction, mean accuracy 93% (V_T_), 96% (RR)	[241]
Multisensor belt	Respiration waveform	Flexible resistive pressure sensor + IMU (MPU6050)	Atmega328P processor, I^2^C ^9^, sensor data fusion (FFT ^10^, STFT ^11^, inertial filtering), eliminates non-breathing motion artifacts, validated vs. spirometry, 6 subjects, RR error < 1 rpm	[242]
ECG ^12^ Holter + wrist IMU	RR, V_T_, Activity	ECG + IMU (Shimmer3)	Activity classification and regression (GLM ^13^, random forest, SVM ^14^, Gaussian process regression, NCA ^15^), 15 subjects, MAE ^16^ 1.17 rpm (RR), 1.39 L/min (V_T_)	[17]
ECG + wrist accelerometer	RR, respiratory waveform	ECG + 3D accelerometer	Reconstruction of respiratory waveform, PSG ^17^ data, signal fusion, validated vs. airflow, 223 subjects, ECG baseline, amplitude, frequency, MAE 0.72 rpm (wrist-motion), 1.08 rpm (chest-motion)	[243]
ECG + accelerometer	RR	ECG + accelerometer	Spectral fusion of EDR ^18^ RSA ^19^ features and accelerometer, adaptive line enhancement based on LMS ^20^, singular spectrum analysis	[244]
Multimodal chest patch	RR	ECG + accelerometer	Cascaded framework for EDR and SDR ^21^, spectrotemporal domain transformation, 2D U-Net denoising, validated vs. COSMED K5, 21 subjects, walking, MAE 0.82 rpm, R^2^ 0.89	[245]
Multimodal chest patch	V_T_	ECG + accelerometer	Fusing EDR + SDR signals via ML ^22^ model, sampling rate 1 kHz, validated vs. COSMED K5, 18 subjects, during activity recovery, RMSE ^23^ 181.45 mL, Pearson correlation coefficient 0.61	[246]
Wrist device	RR	PPG ^24^ + accelerometer	12 subjects on treadmill (walking and running), sampling rate 125 Hz, reconstructs of motion corrupted PPG signals in the Hilbert domain + autoregressive technique, MAE 5.53 rpm	[247]
Finger devices	RR	PPG + accelerometer	MAX30102 PPG sensor, 8 subjects, sitting, validated vs. Vernier chest belt, fusion method, LMS adaptive filter, MAE increased from 3.1 to 1.1 rpm, RR accuracy > 95%	[248]
Smartwatch datasets	RR	PPG + accelerometer	PPG + accelerometer, DL ^25^ method, dilated residual inception modules with multi-scale convolutions, transfer learning, evaluated on PPG-DaLiA and WESAD datasets, MAE 2.29 rpm (PPG-DaLiA)/3.09 rpm (WESAD), RMSE 3.11 rpm (PPG-DaLiA)/3.79 rpm (WESAD)	[249]
Smartwatch	RR	PPG + accelerometer + gyroscope	Samsung Gear Sport watch + Shimmer3 ECG device, DL method incorporating gyroscope data, ulti-scale residual CNN ^26^, evaluated on 1-day recordings, 36 subjects, MAE 1.85 rpm, RMSE 2.34 rpm	[237]
Smartwatch	RR	PPG + accelerometer + gyroscope	LG Urbane watch, sampling rate 20 Hz, validated vs. Zephyr Bioharness 3.0, 14 subjects, IMU rejects corrupted segments, CNN-based RR, tuneable accuracy–latency trade-off, MAE 2.05 rpm (50 s)/1.09 rpm (5 min), 2.5–5.8× lower MAE than prior methods	[250]
Gel-free multimodal chest patch	RR, HR ^27^, HRV ^28^	PPG + accelerometer + temperature	Accelerometer (ADXL355), (500 Hz), PPG (MAX30102), (200 Hz), Atmega328pb, I^2^C, Teager– Kaiser energy operator based SCG ^29^ processing, validated vs. BIOPAC, 12 subjects, daily-life, MAE < 1% (HR), ~1.6% (RR)	[251]
Neck-worn wearable	RR, HR	PPG + accelerometer	22 subjects, guided breathing, RR accuracy: 94.94 ± 3.56% (vs. metronome), 88.4 ± 7.63% (vs. Capnostream), HR accuracy 93.67 ± 7.64%	[252]
Datasets	RR	ECG + PPG fusion	Real-time fusion, 6 derived components filtered by quality index, component analysis, evaluated on Capnobase (n = 42), BIDMC (n = 53), MAE 1.39 rpm (Capnobase)/3.29 rpm (BIDMC), 11.61% average MAE reduction vs. state-of-the-art	[253]
Datasets	RR	ECG + PPG fusion	DWT ^30^, instantaneous signal quality indices used as adaptive fusion weights, evaluated on CapnoBase TBME RR dataset (n = 42), sampling rate 300 Hz, MAE 0.34 rpm, robust across SNR (−50 to 50 dB)	[254]
Dataset	RR, CI ^31^	ECG + PPG fusion	BIDMC (53 subjects), 400 s, sampling frequency 125 Hz, EGPR ^32^ algorithm, adaptive neighbor component analysis, PMF ^33^-EGPR setup MAE 0.98 rpm (CI 4.85 rpm), EMF ^34^-EGPR set. MAE 1.155 rpm (CI 7.47 rpm)	[255]
Multimodal chest patch	RR	ECG + PPG + accelerometer	Respiratory surrogate signals extraction, adaptive channel selection via spectral respiratory quality index, modality-attentive fusion, U-Net-based DL denoising, 17 subjects, MAE 2.21 rpm (walking), MAE reduced to 1.59 rpm (excluding low-quality segments)	[256]
Chest worn sensors	RR, Respiratory waveform	ECG + PPG + IMU	Sampling rate 700 Hz, 15 subjects, evaluated during walking (MAE 2.93 rpm), stair climbing (MAE 3.32 rpm), Bland–Altman mean bias 0.89 rpm (95.2% within LoAs −6.14–7.90 rpm)	[257]
Datasets	RR	ECG + PPG + EMG ^35^ fusion	Capnobase, BIDMC datasets, evaluated LSTM ^36^, CNN–LSTM, and attention-based models, best performance: bidirectional LSTM with attention, MAE 0.24 ± 0.03 rpm (ECG/PPG), MAE 0.51 ± 0.03 rpm (EMG), 64 s observation window significantly improved accuracy vs. 32 s window	[258]
Upper-arm wearable	RR, HR	PPG + Single-sided ECG + BioZ ^37^ + IMU	Microcontroller NucleoWB55RG, sampling rate 100 Hz, 16 subjects, 6 tasks (sitting + controlled breathing + arm movement), multimodal fusion (3× regression model), AM ^38^ + FM ^39^ + BW ^40^ regression, MAE: 0.97 ± 0.62 rpm (Red diode PPG), 0.13 ± 0.27 rpm (BioZ), 0.66 ± 0.88 rpm (EDR), 14-channel regression MAE 0.22 ± 0.37 rpm, BioZ baseline dominated RR estimation (80–95% importance), EDR FM contributed 5–20%.	[43]
Chest worn	RR, V_T_	ECG + SCG + PPG + EMG + BioZ fusion	18 subjects, cycling, V_T_ coefficient of determination 0.91, agreement of respiratory muscle force indices vs. mouth pressure (Spearman ρ = 0.87, repeated measures 0.76)	[260]
Smartwatch	RR, HR	PP + ECG + IMU fusion	Free-living dataset, 46 subjects, multitask DL, MAE 1.98 rpm, RMSE = 2.51 rpm, MAPE = 0.13%	[261]
Chest patch	V_T_	ECG + acoustic	ECG + lung sounds fusion, real-time respiration pattern analysis, 10 mm piezoelectric plate + ECG (RHD2116, Intan Tech Chip), 2.4 GHz communication, different breathing protocols, V_T_ *p*-value 0.0018–0.052	[262]
Chest patch	HR, RR	ECG + acoustic + respiratory sensors	Multi-criterion multimodal fusion ML model, large-scale evaluation (5561 recordings, 475 subjects), 87% accuracy, during exercise, Weight 5.4 g	[264]

^1^ Respiration rate, ^2^ respiratory inductance plethysmography, ^3^ inertial measurement unit, ^4^ Bluetooth low energy, ^5^ limits of agreement, ^6^ recursive least squares, ^7^ signal-to-noise ratio, ^8^ tidal volume, ^9^ inter-integrated circuit, ^10^ fast Fourier transform, ^11^ short-time Fourier transform, ^12^ electrocardiography, ^13^ generalized linear model, ^14^ support vector machine, ^15^ neighborhood component analysis, ^16^ mean absolute error, ^17^ polysomnography, ^18^ ECG-derived respiration, ^19^ respiratory sinus arrhythmia, ^20^ least-mean-square, ^21^ SCG-derived respiration, ^22^ machine learning, ^23^ root mean square error, ^24^ photoplethysmography, ^25^ deep learning, ^26^ convolutional neural network, ^27^ heart rate, ^28^ heart rate variability, ^29^ seismocardiography, ^30^ discrete wavelet transform, ^31^ confidence interval, ^32^ exact Gaussian process regression, ^33^ positive matrix factorization, ^34^ expectation–maximization factorization, ^35^ electromyography, ^36^ long short-term memory, ^37^ bioimpedance, ^38^ amplitude modulation, ^39^ frequency modulation, ^40^ baseline wander.

### 4.6. Conclusions

Multimodal systems represent a key direction for achieving robust respiratory monitoring under real-world conditions. Their advantage lies not in replacing individual sensing modalities but in combining complementary physiological and motion-related information to overcome the limitations of single modalities.

Across the reviewed studies, inertial sensing plays a central role, primarily by providing motion context for artifact suppression and activity-aware adaptation, rather than serving as a standalone modality [238,239,244,247,250]. At the same time, in specific configurations, such as SCG, inertial sensors can also contribute directly to respiratory signal estimation [245,246]. This dual role makes IMUs particularly valuable in multimodal architectures.

The effectiveness of multimodal systems is strongly context dependent. Under stable conditions, relatively simple fusion strategies are often sufficient. However, in ambulatory or dynamic scenarios, performance increasingly depends on signal quality assessment, adaptive channel selection, and fusion strategies, frequently supported by ML. This is particularly evident in wrist-worn devices, where PPG–IMU integration has become essential for maintaining acceptable performance during daily activity [237,249,250].

An important trend is the transition toward fully integrated wearable platforms, including chest patches and textile-based systems. These platforms extend beyond RR estimation and enable broader physiological monitoring, including respiratory waveform reconstruction, V_T_ estimation, and contextual interpretation of movement and posture [17,241,246,262]. However, these improvements come at the cost of increased algorithmic complexity and energy consumption [254,265]. Consequently, the optimal system design depends on the intended application and requires balancing robustness, accuracy, and resource constraints.

Overall, the evidence suggests that multimodal fusion, combined with context-aware processing, currently represents the most practical pathway toward reliable and wearable respiratory monitoring in uncontrolled environments, where single-modality systems remain insufficient.

## 5. Discussion and Conclusions

Modern wearable respiratory monitoring is steadily progressing from proof-of-concept systems toward more integrated, multimodal, and application-oriented solutions.

### 5.1. Review Articles

While the previous sections focused primarily on individual experimental studies and device implementations, several recent review articles provide a broader perspective on wearable respiratory monitoring technologies. These works summarize methodological developments, sensor modalities, and algorithmic approaches across a wider body of the literature, helping to contextualize the findings discussed above. For example, Hussain et al. [18] highlighted the difference between advanced prototypes based on promising materials and commercially available systems, such as Hexoskin and Zephyr, and proposed a frequency-based classification of wearable sensors and emphasized the need to move from laboratory innovations to regulatory-compliant medical products. Their work emphasizes that material innovation alone is not enough without manufacturability, reproducibility, and validation.

A broader perspective at the systems level is provided by Kim et al. [266] and Vicente et al. [267], who extended the discussion beyond RR to multimodal detection of complex biomarkers, IoT connectivity, and ML integration. These studies highlight the growing convergence of mechanical, environmental, and biochemical sensing, as well as the ethical and data governance implications of large-scale respiratory data collection in healthcare ecosystems. Jia et al. [268] and Karpiel et al. [269] further outlined ongoing challenges, including energy efficiency, ergonomic integration, signal stability, and privacy concerns. Non-contact approaches, such as millimeter-wave radars, hold promise for seamless monitoring, especially when combined with AI for predictive analysis. These developments suggest a shift from reactive monitoring to proactive care. Materials innovation remains a central driver of this transformation. Chen et al. [270] and Yin et al. [271] detailed advances in nanomaterials, conductive polymers, and mechanically soft substrates that improve biomechanical compliance and long-term durability. Xu et al. [272] expanded the knowledge on AI-driven soft bioelectronics for self-powered respiratory monitoring. Importantly, Yin et al. also highlighted the emerging frontier of chemical breath analysis, where detection of volatile organic compounds complements mechanical respiration monitoring.

### 5.2. Algorithmic Processing

The transition from raw sensor data to clinically actionable respiratory metrics relies fundamentally on the synergy between hardware design and algorithmic processing [13]. As highlighted throughout this review, hardware innovations alone are insufficient under dynamic conditions. The performance of wearable respiratory monitoring systems is determined not only by the sensing modality itself, but increasingly by the associated signal-processing and algorithmic pipeline. Across most wearable modalities, including chest belts, bioimpedance, IMU/SCG systems, and ECG/PPG-derived respiration, the raw signals are strongly affected by motion artifacts, posture changes, sensor displacement, environmental interference, and inter-subject physiological variability. Consequently, modern respiratory monitoring systems should be regarded as integrated sensor–algorithm platforms rather than isolated hardware solutions.

A fundamental processing stage common to most systems is filtering and signal conditioning. Typically, respiratory signals are bandpass filtered to isolate the breathing-related frequency range while suppressing low-frequency drift and high-frequency noise [16]. Low cutoff frequencies around 0.01 Hz are commonly used to remove slow baseline fluctuations, whereas upper cutoff frequencies are generally below 1 Hz for resting conditions and may extend to 2 Hz in sports or neonatal monitoring applications with elevated RR [7]. Butterworth filters are frequently preferred because of their flat passband characteristics, while Chebyshev Type I filters provide steeper roll-off properties [273]. Following filtering, respiratory estimation algorithms generally divide into time-domain and frequency-domain approaches. Time-domain methods typically rely on peak detection, where RR is derived from the respiratory period between consecutive waveform peaks. Frequency-domain methods based on FFT, Welch’s method for PSD, or periodogram analysis identify the fundamental respiratory frequency [144]. While these classical approaches are computationally lightweight, require minimal memory, and are perfectly suited for edge–device integration [274], their robustness decreases substantially under unconstrained dynamic environments. Simple bandpass filters cannot distinguish between physical movements and respiratory effort if both share the same frequency band, often leading to signal distortion or loss of data during coughs or strenuous exercise [275]. Consequently, wavelet-based denoising and adaptive filtering approaches have attracted increasing interest because they preserve transient respiratory dynamics more effectively.

The importance of sensor–algorithm co-design becomes particularly evident in motion-prone wearable systems. In accelerometer- and strain-based monitoring, motion artifacts often overlap spectrally with the respiratory component. Several studies, therefore, incorporated adaptive filtering driven by auxiliary IMU signals, PCA for dimensionality reduction, or quaternion-based orientation tracking to fuse multi-axis data [276,277]. Madgwick’s algorithm offers a highly favorable balance between computational efficiency, low-sampling-rate operation, and robustness against gyroscope drift, making it ideal for low-power wearables. In these approaches, hardware selection directly constrains the algorithmic possibilities. Adding gyroscopes substantially improves orientation tracking and posture compensation but simultaneously increases power consumption, sampling synchronization requirements, and computational complexity [13].

To address the limitations of classical filters, recent years have seen a substantial shift toward data-driven ML and DL architectures capable of adaptive artifact suppression and nonlinear modality mapping. Supervised models like SVM have been successfully employed for inspiratory/expiratory phase detection using contiguous 4 ms analysis windows [147], while unsupervised learning, such as K-means clustering, has proven critical in grouping morphologically varying SCG events caused by postural changes to reduce waveform heterogeneity prior to RR extraction [153,278]. DL models excel at extracting latent features directly from raw data. For example, recurrent neural networks and meta-learning approaches improve respiratory flow estimation from multimodal FBG and IMU systems, even with reduced sensor counts [279], and mitigate hysteresis and drift in flexible piezoresistive strain sensors, reducing the RMSE by up to 58% [13]. U-Net-based architecture applied to triaxial accelerometer signals significantly improves RR estimation by combining respiratory demodulation and denoising stages [245]. Architectures such as 1D convolutional recurrent neural networks (1D-CRNNs), CNN-LSTM hybrids, ResNet for stride–artifact removal, and transformer-based models increasingly dominate the recent literature because of their superior ability to separate respiratory motion from locomotion artifacts [150,151,152]. The “ResPara-Net” framework demonstrates the efficacy of CNNs in daily activities, achieving normalized MAE below 4% and low RMSE values (0.12–0.14) across various breathing regimes [149]. End-to-end DL models offer superior robustness and can synthesize respiratory waveforms from highly corrupted data, such as extracting RR from wrist-worn PPG during exercise [237,249,250].

Despite high estimation accuracy, DL models face significant translational challenges. Many studies report performance metrics without critically addressing dataset sizes, leading to a high risk of overfitting. A model trained exclusively on stationary subjects or specific postures often lacks generalization capability when deployed in free-living environments. Furthermore, the continuous matrix multiplications required for DL inference impose severe computational and memory demands, resulting in high latency and rapid battery depletion, which limits their real-time feasibility on microcontroller-based wearables [236,245,280]. To improve reliability, several studies have introduced SQI, adaptive channel selection, and context-aware processing pipelines. The concept of sensor–algorithm co-design emphasizes that algorithms should not merely act as post-processing filters but rather dictate how and when hardware operates. SQI algorithms critically evaluate the signal-to-noise ratio in real-time. If the SQI drops below a defined threshold due to severe motion, the system can dynamically power down the primary sensor, switch to an auxiliary modality, or flag the segment to prevent false clinical alarms. Shipper et al. [154] demonstrated this by combining recursive PCA with an SQI threshold, achieving an LoA below 1.45 rpm, with 80% temporal coverage across variable postures. Similarly, in bioimpedance monitoring, identifying and preserving physiologically meaningful segments algorithmically is often as critical as the hardware’s raw sensitivity. However, SQI algorithms must be carefully calibrated, as overly aggressive thresholds may reject valid physiological anomalies like dyspnea, while lenient thresholds allow motion artifacts to corrupt RR estimation.

A consistent conclusion across the reviewed literature is that algorithmic selection is a zero-sum game, balancing accuracy, robustness, and power consumption [236,245,274,280]. Since no single sense of modality performs optimally under all dynamic conditions, modern wearable systems increasingly combine complementary sensing principles. Systematic reviews highlight sensor fusion, such as combining ECG/PPG with IMU context, consistently outperforms complex single-sensor DL models, reducing error rates from 11.9% to 7.3% [280,281]. Fusion may be implemented at the raw-signal, feature, or decision level, each introducing different trade-offs between flexibility, interpretability, synchronization requirements, and computational cost [282,283]. Ultimately, the most effective wearable respiratory systems do not rely on the most complex AI in absolute terms. Instead, they utilize context-aware fusion logic deploying lightweight heuristic algorithms during rest and triggering computationally heavy artifact-suppression ML models only when accelerometers detect significant physical activity [282,283]. Future wearable respiratory systems will therefore likely evolve toward tightly integrated multimodal sensor–algorithm ecosystems optimized jointly for physiological relevance, robustness, energy efficiency, and real-time deployment.

Summary information about the algorithms is written in Table 8.

### 5.3. Limitations of the Validation Protocols and the Need for Metrological Standardization

Despite substantial technological progress in wearable respiratory monitoring, objective comparison across studies remains severely limited. Critical synthesis of the current literature reveals a fundamental metrological issue: studies frequently conflate statistical error with true measurement uncertainty and natural physiological variability. Consequently, the apparent accuracy of many systems often reflects the specific, highly controlled experimental design rather than robust real-world performance. The lack of standardized validation protocols differing in reference methods, breathing tasks, activity types, evaluation windows, and reported metrics significantly limits reproducibility and makes meta-analytical benchmarking or direct ranking practically impossible [284].

Current validation approaches generally fall into three categories: artificial prototypes, metronome-guided breathing, and validation against clinical reference devices. While mechanical prototypes, such as motorized artificial chests [285,286] or custom traction/compression machines for strain sensors [287], eliminate human physiological variability, they fail to replicate the complex morphological variations of human respiration. Conversely, metronome-guided validation introduces human compliance errors, as subjects rarely follow the acoustic pacing perfectly. Validation against reference devices (e.g., capnography or spirometry) is the most clinically relevant method, but it introduces a major, often unacknowledged bias: the reference devices themselves possess inherent measurement uncertainties and synchronization delays that are rarely quantified or factored into the final error evaluation.

A particularly important source of variability is the breathing pattern itself. Respiratory monitoring performs best under paced breathing, where the waveform is well defined and quasi-periodic. Under these stationary conditions, many algorithms naturally achieve excellent apparent accuracy. In contrast, during spontaneous, irregular, or conversational breathing, the signal becomes highly susceptible to interference from posture changes, speech, sensor displacement, and motion artifacts, which can significantly degrade sensor performance [288,289]. This likely explains why accuracy often appears very high at rest, deteriorates significantly during moderate unconstrained activity, and partially stabilizes again at higher exercise intensities where breathing mechanically returns to a rhythmically constrained pattern [284].

From a metrological perspective, a significant shortcoming in the existing literature is the lack of adherence to standardized frameworks, such as the ISO Guide to the Expression of Uncertainty in Measurement (GUM) [290]. Most studies report discrete error metrics, such as the absolute error, MAE, or RMSE, without defining measurement uncertainty, repeatability, or traceability to calibrated standards.

Furthermore, the reported metrics are highly inconsistent. While some authors report accuracy as a percentage of correctly identified patterns, others rely on linear regression, correlation coefficients, or purely descriptive statistics. Even when Bland–Altman analysis [291] is employed to assess agreement, studies frequently report only the mean bias, omitting the LoA, which is crucial for understanding the true variability and measurement envelope of the sensor under test. In fact, only a limited number of studies currently provide comprehensive statistical evaluations that combine LoAs with complementary metrics like MAE or correlation factors [292,293,294]. Without these standardized bounds, distinguishing whether a variation is caused by sensor inaccuracy, algorithmic limitation, or true physiological change is impossible.

Another critical but frequently underreported factor is the evaluation window length. Longer time windows inherently smooth out random variability and improve the stability of the estimated RR. Across the reviewed literature, reported accuracy often increases with window duration, frequently saturating around 30 s. Therefore, the choice of window length is not a minor technical detail, but a core design parameter that biases the reported accuracy. Furthermore, the current literature is heavily skewed toward short-term laboratory validation. The long-term behavior of these sensors, including the effects of temperature drift, material aging, prolonged wear, and continuous dynamic motion, remains largely unexplored, highlighting a significant gap in evaluating long-term reproducibility.

From a translational perspective, the field would benefit substantially from adopting strict metrological validation recommendations. Future studies should explicitly distinguish between systemic bias, random error, and physiological variability. To enable future quantitative meta-analyses, authors should standardize their reporting by:

- Providing full Bland–Altman statistics (mean bias and 95% LoA) alongside MAE or RMSE.
- Clearly defining calibration procedures, synchronization techniques, and the inherent measurement uncertainty of the chosen reference device.
- Standardizing evaluation windows and testing protocols to include both paced rest and unconstrained dynamic activities.
- Evaluating the agreement of the reconstructed respiratory waveform itself (or a standardized periodic surrogate), rather than solely comparing the extracted RR, to better reflect the physiological fidelity of the output. Obtaining an accurate estimate of deeper volumetric parameters remains a significant challenge, especially for wearable systems, with only a limited number of studies addressing it simultaneously.

### 5.4. Comparative Performance Analysis

Given the previously discussed limitations of direct statistical aggregation, we propose a synthesis based on approximate performance envelopes and a comparative methodological matrix. Instead of directly ranking technologies using often incompatible accuracy metrics, this framework emphasizes the practical trade-offs between motion robustness, wearability, computational complexity, and suitability for estimating specific respiratory parameters such as RR and V_T_.

Although exact error values vary depending on datasets and validation protocols, several general performance trends can still be identified. Under controlled resting conditions, differences between sensing modalities become relatively small. Both indirect approaches and direct methods, bioimpedance, and chest belts commonly achieve MAE between 0.5 and 2.0 rpm, while LoAs usually remain below 3 rpm. Under moderate and high-intensity dynamic conditions, however, the performance envelopes diverge considerably due to differing susceptibility to motion artifacts. Wrist-worn PPG systems using conventional processing show the largest degradation, where errors can increase to 4–6 rpm or more because rhythmic arm motion often overlaps with respiratory modulation. Chest-mounted ECG-derived respiration generally maintains lower errors, typically around 2–4 rpm, although severe baseline wander caused by electrode motion during exercise remains a major limitation. Direct thoracic sensing approaches, particularly chest belts and bioimpedance systems, provide more stable performance due to their stronger mechanical coupling with thoracic expansion, usually maintaining errors around 2–3 rpm. The best performance in dynamic scenarios is currently achieved by multimodal fusion systems integrating PPG or ECG with IMUs and AI-assisted processing. By combining sensor redundancy with adaptive artifact compensation, these systems can reduce errors to approximately 1–2.5 rpm, approaching resting-state performance.

As summarized in Table 9, wearable respiratory monitoring inherently involves a trade-off between wearability and signal robustness. Wrist-worn PPG devices offer the highest comfort and user compliance, requiring no additional hardware and relatively low energy consumption, but their robustness during movement remains limited, and V_T_ estimation is generally not feasible. Conversely, chest belts and strain-based systems provide physiologically interpretable signals with higher robustness, although reduced comfort limits their suitability for long-term continuous monitoring.

Overall, the most promising compromise currently appears to be flexible multimodal chest patches combining bioimpedance, ECG, SCG, or chest belts with IMU sensors and adaptive ML algorithms. These systems achieve relatively high robustness while preserving acceptable wearability. Their main limitation shifts toward increased computational and energetic demands, algorithmic complexity, and the frequent need for subject-specific calibration.

## Figures and Tables

**Table 8:** Summary and comparative analysis of algorithmic frameworks.

Algorithm Category	Typical Methods	Estimation Accuracy	Comput. Complex.	Power Consum.	Real-Time Feasibility	Key Limitations
**Classical signal** **processing**	Digital filters, zero-crossing, peak detection	Moderate (static)/poor (dynamic)	Low	Low	Yes (basic µcontrollers)	Susceptible to artifacts, unable to separate overlapping motion/breathing frequencies
**Adaptive filtering & decomposition**	PCA ^1^, wavelet transforms, Madgwick algorithm, SQI ^2^	Good (improved artifact handling and posture stability)	Low to medium	Low to medium	Yes (edge devices)	Sensitive to posture changes, requires rigorous heuristic parameter tuning
**Machine learning**	SVM ^3^, K-means clustering, Gaussian process regression	High (in constrained scenarios)	Medium	Medium	Edge AI	Generalization challenges, risk of overfitting specific training datasets or postures
**Deep learning**	U-Net, ResNet ^4^, 1D-CRNN ^5^, CNN-LSTM ^6^, diffusion models	Very high (low MAE ^7^)	High	High	Edge TPU ^8^ (cloud required)	High latency, extensive memory constraints, rapid battery depletion, “black-box” interpretability
**Multimodal** **fusion**	MTL ^9^, adaptive SQI-gating, cross-sequence mapping	Excellent (robust in dynamic)	High	Medium to high	Edge AI (context-aware execution)	High integration complexity, requires sensor synchronization and calibration

^1^ Principal component analysis, ^2^ signal quality indices, ^3^ support vector machines, ^4^ residual network, ^5^ one-dimensional convolutional recurrent neural network, ^6^ convolutional neural network long short-term memory, ^7^ mean absolute error, ^8^ tensor processing unit, ^9^ multitask learning.

**Table 9:** Comparative methodological matrix of wearable respiratory monitoring approaches.

Principle + Algorithm	Typical Parameter	Motion Robust ^1^	Comfort	Energy	Comp. compl ^2^	Pers ^3^	Advantages	Limitations
**Chest belt + classical** **filtering**	RR ^4^, limited V_T_^5^	Mid	Mid	Low	Low	Low	Simple implementation, high physio interpretability	Motion artifacts, discomfort, limited long-term compliance
**Flexible, patches + classical** **filtering**	RR, limited V_T_	Mid- high	Mid- high	Low	Low	Low	Simple implementation, high physio interpretability, increased comfort	Durability, calibration to individual body type
**Chest belt + adaptive/ML ^6^**	RR, improved V_T_	Mid- high	Mid	Mid	Mid	Mid-high	Improved drift correction, better robustness	Increased energy and computational load, limited long-term compliance
**BioZ ^7^ + adaptive/ML**	RR, V_T_	Mid- high	Mid	Mid	Mid	Mid-high	Direct link to lung volume—V_T_, ECG ^8^ compatible	Sensitive to skin– electrode impedance, requires calibration, EMG ^9^ artifacts
**EDR ^10^ +** **regression/ML**	RR, HR ^11^	Low- mid	Mid (patch)	Mid	Mid	Mid-high	No extra hardware, interpretable features	Sensitive to electrode placement and motion
**PPG ^12^ + classical signal** **processing**	RR, HR	Low	High	Low- mid	Low	Low	Easily embedded, low hardware complexity	Sensitive to motion, limited V_T_ estimation capability
**PPG + ML (IMU ^13^** **fusion)**	RR, HR, limited V_T_	Mid- high	High	Mid- high	Mid- high	High	Improved artifact compensation, nonlinear modeling	Higher computational cost, model generalization challenges
**SCG ^14^/IMU + signal** **decomp ^15^**	RR, HR, limited V_T_	Mid	Mid-high (patch)	Low-Mid	Mid	Mid	Simultaneous cardiorespiratory mechanics	Requires signal separation, posture sensitivity
**Multimodal fusion + ML/DL ^16^**	RR, HR, improved V_T_	High	Mid	High	High	High	Redundancy, artifact compensation, robustness	Hardware complexity, higher power, needs calibration

^1^ Motion robustness, ^2^ computational complexity, ^3^ personalization, ^4^ respiration rate, ^5^ tidal volume, ^6^ machine learning, ^7^ bioimpedance, ^8^ electrocardiography, ^9^ electromyography, ^10^ ECG-derived respiration, ^11^ heart rate, ^12^ photoplethysmography, ^13^ inertial measurement unit, ^14^ seismocardiography, ^15^ decomposition, ^16^ deep learning.

## Data Availability

No new data were created or analyzed in this study.

## Abbreviations

The following abbreviations are used in this manuscript:

1D-CRNN	One-dimensional convolutional recurrent neural network
AdaBoost	Adaptive Boosting
ADC	Analog-to-digital converter
AFE	Analogue front-end
AGTBO	Advanced golden tortoise beetle optimizer
AI	Artificial intelligence
A-LSTM	Attention-based long short-term memory
ANC	Adaptive noise cancellation
ANCA	Adaptive neighbor component analysis
AUC	Area under the curve
BioZ	Bioimpedance
bpm	Beat per minute
CAM	Count advanced method
CMRR	Common-mode rejection ratio
CNN	Convolutional neural network
CNTs	Carbon nano tubes
COPD	Chronic obstructive pulmonary disease
CPET	Cardiopulmonary exercise test
CT	Computed tomography
DFT	Discrete Fourier transformation
DNN	Dense neural network
DL	Deep learning
DWT	Discrete wavelet transform
ECG	Electrocardiography
EDR	ECG-derived respiration
EEG	Electroencephalography
EGPR	Exact Gaussian process regression
EIP	Electrical impedance plethysmography
EIT	Electrical impedance tomography
ELM	Extreme learning machine
EMD	Empirical mode decomposition
EMG	Electromyography
EMI	Electromagnetic interference
ESD	Electrostatic discharge
EWMA	Exponentially weighted moving average
FBG	Fiber Bragg gratings
FFT	Fast Fourier transform
FIFO	First-in, first-out
FIR	Finite impulse response
GAN	Generative adversarial network
GPR	Gaussian process regression
GNS	Graphene nanosheet
HIIT	High-intensity intermittent training
HR	Heart rate
ICC	Intraclass correlation coefficient
ICU	Intensive care unit
IMU	Inertial measurement unit
IoT	Internet of Things
IPSG	Imbalanced power spectral generation
LCCC	Lin’s concordance correlation coefficient
LMSs	Least mean squares
LoAs	Limits of agreement
LOSO	Leave one subject out
LSTM	Long short-term memory
MAE	Mean absolute error
MAPE	Mean absolute percentage error
mCAFT	Modified Canadian aerobic fitness test
MEMSs	Microelectromechanical systems
ML	Machine learning
MLP	Multilayer perceptron
MMFE	Multiple multilevel feature extraction
MMG	Mechanomyography
MSE	Mean squared error
OSA	Obstructive sleep apnea
PCA	Principal component analysis
PCG	Phonocardiogram
PDMS	Polydimethylsiloxane
PET	Polyethylene terephthalate
PPG	Photoplethysmography
PSG	Polysomnography
PVDF	Polyvinylidene fluoride
RIP	Respiratory inductance plethysmography
RLSs	Recursive least squares
RMSE	Root mean square error
RNN	Recurrent neural network
rpm	Respiration per minute
RR	Respiration rate
RSA	Respiratory sinus arrhythmia
SCG	Seismocardiography
SDR	SCG-derived respiration
SEC	Segregated envelope and carrier
SEM	Standard error of measurement
SHAP	Shapley additive explanation
SNNs	Spiking neural networks
SNR	Signal-to-noise ratio
SQA	Signal quality-aware
SQI	Signal quality index
STFT	Short-time Fourier transform
SVM	Support vector machine
TENG	Triboelectric nanogenerator
VE	Minute ventilation
V_T_	Tidal volume
WBSN	Wireless body sensor network
